# Revising evidence of hurricane strikes on Abaco Island (The Bahamas) over the last 700 years

**DOI:** 10.1038/s41598-020-73132-x

**Published:** 2020-10-06

**Authors:** Tyler S. Winkler, Peter J. van Hengstum, Jeffrey P. Donnelly, Elizabeth J. Wallace, Richard M. Sullivan, Dana MacDonald, Nancy A. Albury

**Affiliations:** 1 Department of Oceanography, Texas A&M University, College Station, TX 77840 USA; 2 Department of Marine Sciences, Texas A&M University At Galveston, Galveston, TX 77554 USA; 3 Department of Geology and Geophysics, Woods Hole Oceanographic Institution, Woods Hole, MA 02543 USA; 4 Massachusetts Institute of Technology/Woods Hole Oceanographic Institution Joint Program in Oceanography, Woods Hole, MA 02543 USA; 5 Department of Geosciences, University of Massachusetts-Amherst, Amherst, MA 01003 USA; 6 National Museum of The Bahamas, PO Box EE-15082, Nassau, Bahamas

**Keywords:** Natural hazards, Palaeoceanography, Palaeoclimate

## Abstract

The northern Bahamas have experienced more frequent intense-hurricane impacts than almost anywhere else in the Atlantic since 1850 CE. In 2019, category 5 (Saffir-Simpson scale) Hurricane Dorian demonstrated the destructive potential of these natural hazards. Problematically, determining whether high hurricane activity levels remained constant through time is difficult given the short observational record (< 170 years). We present a 700-year long, near-annually resolved stratigraphic record of hurricane passage near Thatchpoint Blue Hole (TPBH) on Abaco Island, The Bahamas. Using longer sediment cores (888 cm) and more reliable age-control, this study revises and temporally expands a previous study from TPBH that underestimated the sedimentation rate. TPBH records at least 13 ≥ category 2 hurricanes per century between 1500 to 1670 CE, which exceeds the 9 ≥ category 2 hurricanes per century within 50 km of TPBH since 1850 CE. The eastern United States also experienced frequent hurricanes from 1500 to 1670 CE, but frequency was depressed elsewhere in the Atlantic Ocean. This suggests that spatial heterogeneity in Atlantic hurricane activity since 1850 CE could have persisted throughout the last millennium. This heterogeneity is impacted by climatic and stochastic forcing, but additional high-resolution paleo-hurricane reconstructions are required to assess the mechanisms that impact regional variability.

## Introduction

**Figure 1:**
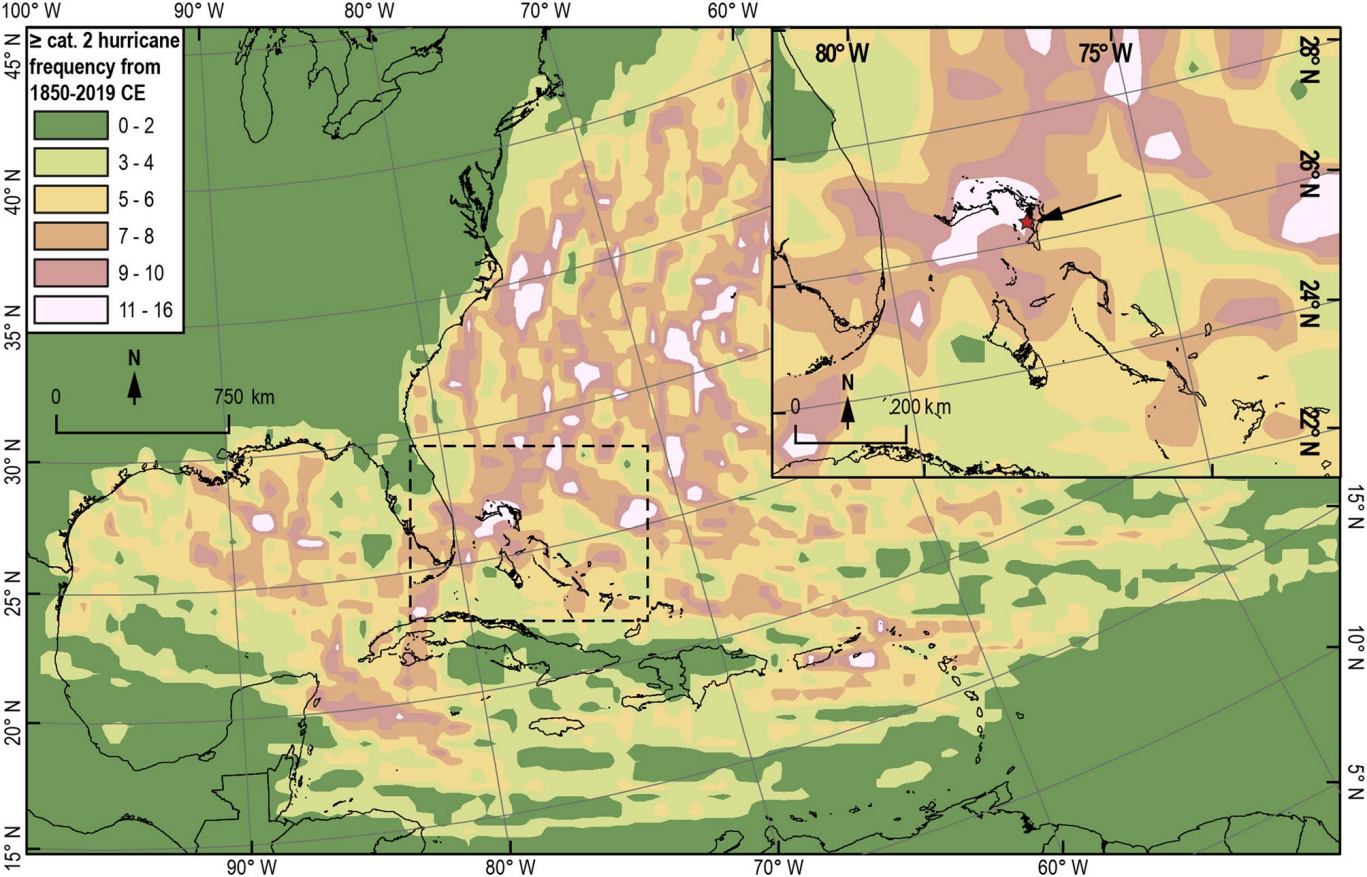
Frequency of exposure to ≥ category 2 hurricane winds (Saffir-Simpson scale) within a 50 km radius from 1850 to 2019 CE throughout the North Atlantic. Storm track and intensity data is from the International Best Track Archive for Climate Stewardship (IBTrACS) v04^1, 2^dataset. Saffir-Simpson storm intensity is specifically derived from the IBTrACS v4 subset USA_Agency_SSHS, which is derived from HURDAT_ATL maximum wind speed data. TPBH is indicated with a red star pointed out by the black arrrow in the inset panel. The regions that we refer to as modern hurricane hotspots are pinkish-white in color (11–16 ≥ category 2 events within 50 km). The Little Bahama Bank is one of these hurricane hotspots. Basemaps were downloaded from *DIVA-GIS *^73^*.* Map generation and associated spatial calculations were performed in ArcMap 10.7.1 software using North America Albers Equal Conic Area projected coordinate system^74^. See SUPPLEMENTAL 1 for detailed methods on how the map was created.

**Figure 2:**
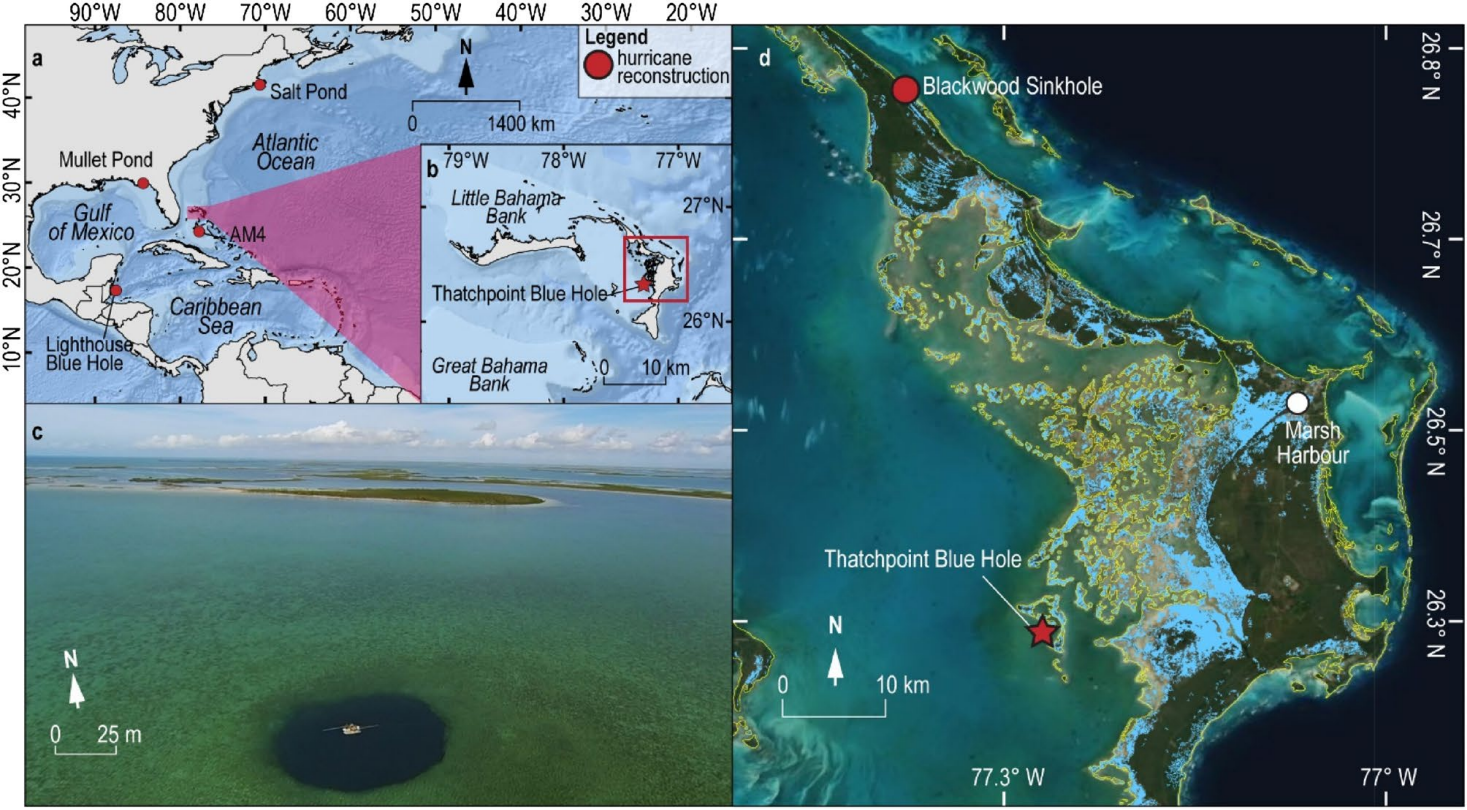
Thatchpoint Blue Hole regional context. (**a,b**) Location of TPBH (red star) in the North Atlantic, as well as the location of some huricane reconstructions cited in this study (red circles, Basemap source: *Esri Oceans*). (**c**) Aerial photograph of TPBH collected by Pete van Hengstum in January 2015 using a DJI Phantom 3 with a mounted GoPro Hero3 digital camera. (**d**) NASA ARIA satellite flood proxy map of Abaco Island on September 4th, 2019 during the peak flooding of Hurriane Dorian (cat. 5, Saffir-Simpson)^39^. The general terrestrial boundaries for Abaco are indicated in yellow (*DIVA-GIS*)^73^, and Dorian induced flooding is shown in light blue. Optical satelitte imagery was obtained from *ESRI* basemap compiled satellite imagery.

The northern Bahamas has been a hurricane hotspot during the observational period (1850 CE to present) with some of the highest incidence of hurricane passage in the North Atlantic Ocean (~ 13 ≥ category 2 events within 50 km of any point in the northern Bahamas, Fig. 1) These storms primarily formed in the Main Development region (MDR)^1–3^ before tracking westward to the northern Bahamas (Fig. 4b–e), causing devastating wind and flood damage to Abaco Island and Grand Bahama Island, before they ultimately track northwards into higher latitudes (Figs. 2, 4b-e)^1, 2^. On 26 August 2019, for example, Hurricane Dorian made landfall on Abaco Island at category 5 strength (sustained winds up to 296 km hr^-1^) and caused widespread economic and human loss. Future hurricane risk assessments mostly rely on observations taken over the last 170 years. However, it remains unclear whether the high rate of hurricane passage through the northern Bahamas has persisted through time or is simply an artifact of stochastic randomness from this short dataset.

Models that evaluate hurricane activity in response to 2 °C global warming indicate a medium-to-high confidence level that the global proportion of intense hurricanes (category 4–5) will increase^4–10^. This trend toward more frequent intense hurricanes has already been observed in a statistical assessment of hurricanes recorded from 1979–2017^11^. Four of the costliest Atlantic hurricanes during the last 170 years occurred between 2012 and 2019 CE (up to USD $336 billion in damage^12, 13^; Sandy-2012, Harvey-2017, Irma-2017, Maria-2017), demonstrating the socio-economic threats posed by more frequent intense hurricanes. Disentangling natural hurricane variability versus the impact of anthropogenic carbon dioxide loading of the atmosphere on future hurricane activity remains a significant climatological forecasting challenge for the twenty-first century. The northern Bahamas emerges as a key geographic locality to assess the natural hurricane patterns over the past millennium given the high occurrence of recent hurricane passage (Fig. 1), and the potential for local geological records to document meaningful change in long-term hurricane activity.

Paleo-hurricane studies can assess background levels of hurricane activity at a given location, at least for the more intense storms. Many of these studies have identified dramatic variability in hurricane activity over the past few thousand years with multi-decadal to centennial-scale active and quiet intervals. Furthermore, these reconstructions are useful for anticipating future hurricane activity because the simultaneous influence of climate on hurricane activity at multiple sites can be assessed. Prehistoric hurricane landfall frequency has been reconstructed using hurricane-induced sediment deposits in salt-marshes^14, 15^, back-barrier coastal ponds^16–21^, and deep-sea sediments^22^. In the last decade, however, the stratigraphy preserved in blue holes and sinkholes on carbonate platforms has emerged as providing faithful archives of paleo-hurricane passage^3, 23–29^. Blue holes form when dissolution of underlying carbonate bedrock forms a void or cave so large that the ceiling cannot sustain its own weight and collapses^30^. The stratigraphic architecture in a blue hole is often well preserved due to negligible exposure to waves or currents and bottom water dysoxia^25^.

Several paleo-hurricane reconstructions spanning the last several millennia at different resolutions (multi-decadal to annual), have linked latitudinal variability in Atlantic hurricane strikes to patterns of sea surface temperature variability in the tropical North Atlantic Ocean and the position of the Intertropical Convergence Zone^3, 25^. This result is consistent with some climate models^31^. In addition, paleo-hurricane activity in the sub-basins of the North Atlantic Ocean (e.g., Gulf of Mexico^27, 29^, Caribbean^23, 32^, U.S. East Coast^18^) exhibits spatial heterogeneity, based on the emerging array of high-resolution paleo-hurricane studies (< 5-yr resolution per sample, spanning > 500 years duration). Additional high-resolution paleo-hurricane studies are required to evaluate the degree to which this spatial heterogeneity is caused by stochastic variability versus the ocean-climate system.

Here we document that Great Abaco Island on the Little Bahama Bank experienced at least 18 intense hurricane strikes (*minimum* estimate, based on uncertainties) from 1500 to 1670 CE by developing a near annually-resolved record of hurricane passage from the sediment preserved in Thatchpoint Blue Hole (TPBH). This period from 1500 to 1670 CE (duration: 170 years), documents a minimum level of hurricane activity that exceeds the total number of ≥ category 2 observed events that passed near TPBH from 1850 to 2017 CE (duration: 169 years, 13 events in IBTrACS^1, 2^). This establishes a clear precedence for higher prehistorical hurricane activity in the northern Bahamas, and these results are critical to help diagnose climate influences on spatial patterns of intense hurricane activity through time.

## Study site: Thatchpoint Blue Hole

### Regional setting

TPBH is ~ 30 km southwest of Marsh Harbour on Great Abaco Island on the leeward margin of the Little Bahama Bank, which is the northernmost carbonate platform in the Bahamian Archipelago (26.32°N, 77.29°W, Fig. 2a, b). TPBH is currently 70 m deep and 50 m wide^24^. Antecedent lithology of this platform is ~ 10 km of mostly shallow-water carbonates that began accumulating in the Jurassic^33^. Weathering and erosion has since dissolved these shallow-water carbonates into a mature karst landscape with abundant caves, terrestrial sinkholes, and flooded blue holes^30, 34^. TPBH is located at the transition between the Bight of Abaco (a shallow, subtidal lagoon) and the carbonate tidal flats on the western island margin (Fig. 2c). Temperature and salinity vary seasonally in the Bight of Abaco from 20ºC to 30ºC and 34 to 39 psu, with tidal pumping and wind-driven vertical mixing maintaining a stable tropical carbonate lagoon^35^. The benthos in the Bight of Abaco is covered with sandy-mud carbonate sediment that is mobilized during storm events, with the preferential off-bank export of finer particles as suspended load^36^. Based on previous regional observations^36^, we expect that high waves, surge and current velocities during hurricane events mobilize and resuspend benthic sediment in the Bight of Abaco. Such physical processes are expected to naturally promote sand-layer accumulation in a natural settling tube, like a blue hole, following hurricane passage (i.e., Thatchpoint Blue Hole). Local sedimentary environments have provided ample sediment supply for transport into TPBH during the last millennium, since the Bight of Abaco became a subtidal carbonate lagoon by ~ 5500 cal yrs BP^37^ (calibrated years before present ) and the carbonate tidal flats formed by ~ 2000 cal yrs BP^38^. Based on satellite imagery after Hurricane Dorian in September 2019^39^, the western margin of Abaco Island where TPBH is located is vulnerable to hurricane surge and flooding (Fig. 2d).

### Previous hurricane record from Thatchpoint Blue Hole

**Figure 3:**
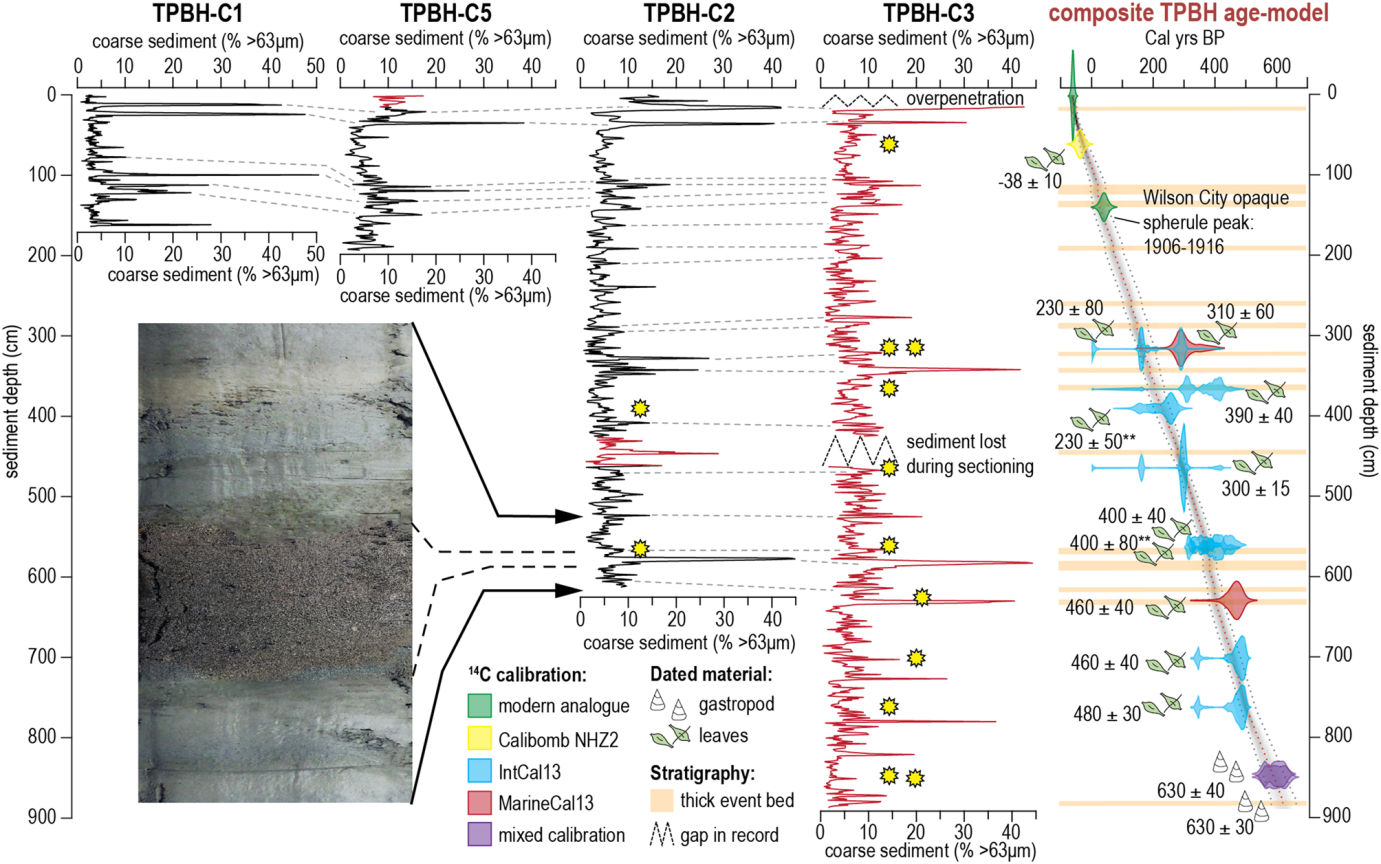
Preserved sedimentary archives, stratigraphic correlation, and sedimentary age-model. Downcore sediment textural data (% D_>63 µm_) for all four cores from TPBH, with TPBH-C1 data derived from van Hengstum, et al.[^24^]. The red lines are the portion of each individual core that was utilized in the composite TPBH record. Coarse sediment layers like the one in the photograph are generally considered to be hurricane event deposits. As indicated by the grey dashed lines, correlation of peaks is well maintained across the cores. The composite TPBH age-model was constructed using 15 points of age control including radiocarbon dated gastropods and terrestrial leaves from TPBH-C3 (*n* = 11 dates) and C2 (*n* = 2 dates), opaque spherule abundance, and modern core-top attribution of 2015 CE. The location of radiocarbon dates within each core is indicated by a yellow star.

In May 2011 (prior to Hurricane Irene in August 2011), SCUBA divers collected a 164 cm core (TPBH-C1, Fig. 3) from TPBH to evaluate the potential of its stratigraphy to document paleo-hurricane activity^24^. The core indicated that background sedimentation in TPBH was fine to medium carbonate silt (15–25 µm) juxtaposed with coarser-grained layers (mean grain size > 45 µm). No terrestrial leaves were found in the stratigraphy, so radiocarbon dating of bivalves (i.e., *Barbatia domingensis*), ^210^Pb, and ^137^Cs provided age control. The radioisotopes indicated (^14^C, ^210^Pb, and ^137^Cs) that the upper 60 cm of TPBH-C1 were deposited between 1950 and 2011 CE, with an average sedimentation rate of ~ 1.6 cm yr^-1^. The uppermost coarse-grained (sand) sedimentary layers were attributed to Hurricane Jeanne in 2004 (category 3) and Hurricane Floyd in 1999 (category 3).Together, the previously described sedimentary processes in the Bight of Abaco^36^, and the preserved stratigraphy in TPBH make this a suitable location to evaluate paleo-hurricane passage.

Problematically, the radiocarbon dated *Barbatia domingensis* shells in the lower part of TPBH-C1 (61 to 164 cm) suggested that the sedimentation rate of 1.6 cm yr^-1^ (0 to 61 cm) abruptly decreased at ~ 1950 CE to ~ 0.1 cm yr^-1^ (61 to 164 cm). As discussed below, the new longer sediment cores collected from the center of TPBH do not contain *B. domingensis* shells, and the cores document a constant sedimentation rate in TPBH (1.3 cm yr^-1^) spanning the last 700 years using an age model built with plant macrofossils. Elsewhere, *B. domingensis* lives attached to reefs and underwater cave walls, and the shell experiences post-mortem detachment and incorporation into the sediment record^40, 41^. Three relevant points emerge: (i) it is likely that TPBH-C1 was collected from a cave area (i.e., underneath a ceiling, Supplementary Fig. S2), (ii) the bivalves that were dated from lower in the core of TPBH-C1 became detached from the ceiling and incorporated into the sediment record long after their death, and (iii) the original 164 cm of stratigraphic sampled by TPBH-C1 actually preserves a continuous record of ~ 100 years (~ 1910 to 2011 CE), not 1000 years as previously reported^24^.

## Results

### Sediment record & age control

New sediment cores sampling the upper 888 cm of the sediment were collected from the center of TPBH (Supplementary Fig. S2). As with previous work, background sedimentation is marine carbonate mud (2.5Y 7/1, clay- to silt-sized particles) with fine-grained white, tan, and green laminations, with inclusions of small intertidal gastropods (e.g., *Cerethium* sp.) and subtidal marine benthic foraminifera (e.g., *Peneroplis sp.*). The laminations and bedding indicate no mechanical mixing of the sediment by wave action or bioturbation. Interrupting the marine carbonate mud is coarse-grained carbonate sand (> 63 um) deposits that are devoid of internal bedding and often dark grey (2.5Y 5/1). The mean coarse sediment fraction (%D_>63 µm_; % mass (mg) of > 63 µm particles per sampled sediment mass) in TPBH-C3 is 6.84% (range: 0.36 to 44.18%, Fig. 3). Qualitative assessment of the particles in these coarse beds shows that they often contain fragmented macroinvertebrate skeletal material and larger (pebble-sized) clasts of weathered karst. Sediments surrounding TPBH in the subtidal carbonate lagoon have been characterized primarily as muddy sand^36^, but karst fragments do occur on the proximal tidal sand bars^42^.

TPBH-C3 and TPBH-C2 have overlapping stratigraphy for 612 cm for assessing the reproducibility of the sedimentary signals. By visually evaluating the coarse sediment anomalies (CSA) that exceed that event-bed definition threshold of 4.39% D_>63 µm_ CSA in TPBH-C3 and TPBH-C2 (see METHODS), it qualitatively appears that coarse-grained sedimentary bed incidence is replicated by each core (Fig. 3). TPBH-C3 records 34 events while TPBH-C2 records 33 events, when the same quantitative methodology for event threshold calculation is applied (described in METHODS). The stratigraphic coherency of overlapping intervals of TPBH-C3 and TPBH-C2 lends confidence toward using the equivalent stratigraphic section from TPBH-C2 to “fill” the gap that occurred during sectioning of TPBH-C3 (426–463 cm, Fig. 3, see METHODS) to create a compiled composite record which records 53 total events in 888 cm.

The composite TPBH record has a near linear sedimentation rate of 1.3 cm yr^-1^ spanning the last ~ 700 years. This is based on an assessment of a fossil fuel combustion chronohorizon between 1906 and 1916 CE, and 13 radiocarbon dates calibrated into sidereal years from both TPBH-C3 (*n* = 11) and TPBH-C2 (*n* = 2) (see METHODS, Supplementary Table S1, SUPPLEMENTAL 3). This means that each 1 cm sample represents ~ 0.77 years of time (i.e., sub-annual resolution), with an average age- uncertainty of ± 24.4 yrs cm^-1^. This is within uncertainties of ~ 1.6 cm yr^-1^ estimate derived for the last 66 years based on post-1950 CE radiocarbon dates and ^210^Pb activity in TPBH-C1^24^. The basal dates for the composite record come from two radiocarbon dated *Cerethium sp.* gastropod shells from TPBH-C3 (METHODS), which were calibrated with a mixed 50/50% calibration curve (IntCal13 and Marine13^43^) to reflect the incorporation of both marine- and terrestrially-derived carbon into the production of their biogenic carbonate. This assumption produces a calibrated 2σ age range of 585 ± 50 cal yrs BP and 589 ± 50 cal yrs BP for these two shells. This is within uncertainties of the resultant stratigraphic age produced by a sedimentation rate of ~ 1.3 cm yr^-1^, if only the first 13 points age-control are considered.

The top 60 cm of the composite record has the least age uncertainty, whereby each 1 cm of sediment deposition has an average age uncertainty of ± 12 years. This interval is largely constrained by a post-bomb radiocarbon date from a leaf at 61.5 cm (1983 to 1992 CE) and the fossil fuel combustion chronohorizon at 140 cm (aged 1906 to 1916 CE) interpreted from a peak in opaque spherule concentration (370 spherules/cm^3^, Supplementary Table S2). The age-model during the historic period is independently supported by downcore ^137^Cs activity (see SUPPLEMENTAL 3, Supplementary Fig. S4). The highest age uncertainties exist at the base of the composite record (800 to 888 cm), where each centimeter has an average 2σ uncertainty of ± 50 years. The entire composite record (888 cm) spans ~ 1330 CE to 2014 CE, with the topmost 225 cm of sediment preserving the 164-year observational period (1850 to 2014 CE).

## Discussion

### Historical hurricane strikes: calibrating the record

An upper threshold of > 4.39% D_>63 µm_ CSA in the quantitative textural data was calculated as indicating a coarse-grained sedimentary layer that is possibly anomalous and a disruption to background blue hole sedimentation (METHODS). Eleven layers (or event beds) meet this threshold over the last 164 years (i.e., historical + instrumental period, E1 to E11), with 53 total event beds since 1330 CE. In paleotempestology, stratigraphic successions are evaluated for textural evidence of long term hurricane passage, with the highest quality reconstructions documenting only hurricane events. As such, other mechanisms that generate coarse sediment anomalies in the stratigraphic record like tsunamis or earthquakes must also be considered^44–46^. However, it is highly unlikely that tsunamis or earthquakes generated tempestites in TPBH because the northern Bahamas are positioned along a passive North American margin that has likely been tectonically stable during the Quaternary period^47^, and there is no record of significant tsunami impacts in this region in recent history^48, 49^. For example, maximum modeled wave runup during the 1755 CE earthquake in Lisbon, Portugal is only ~ 0.75 m in far field locations like on the eastern margin of the northern Bahamas^50^.

Several sources of uncertainty must be considered when attributing coarse-grained sedimentary deposits to individual hurricane events, including possible changes in hydrodynamics from individual storms and how those hydrodynamics interact with local sediment budgets and coastal landforms. It is likely that hurricanes of varying characteristics (i.e., intensity, track, size, translation speed) can generate event beds at TPBH. At Apalachee Bay in Florida, for example, hydrodynamical modeling indicates that although proximal category 4 and 5 storms produced the most extreme storm surge, medium intensity category 1–3 hurricanes are also capable of producing extensive local storm surge as a result of larger inner wind fields, storm track translational speed, or proximity^51^. Sedimentologically, stratigraphic undercounting of hurricane events likely occurs during periods of frequent hurricane passage, where a single coarse grained layer may represent more than one hurricane event^52^ (i.e., time averaging). Advantageously, the high frequency of hurricane passage near TPBH since 1850 CE (Fig. 1) and archival of 11 event beds during the same period can be compared to evaluate the potential for different storm categories to generate event beds in TPBH.

**Figure 4:**
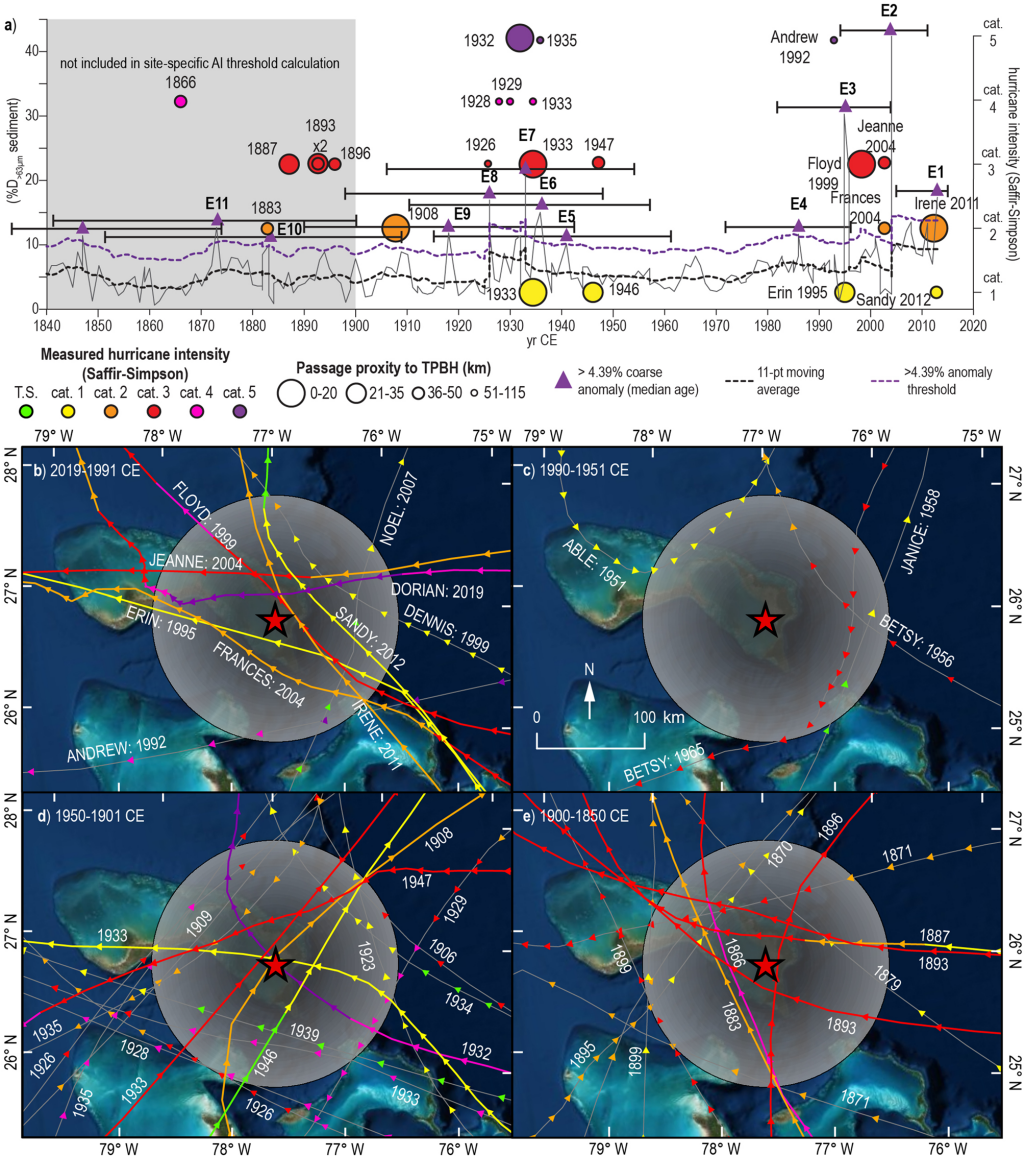
Sediment record sensitivity calibration/attribution to IBTrACS observational hurricane record from 1850 to 2014 CE. a) Coarse sediment (%D_>63 µm_) plot (grey line) from TPBH composite record from 1850 to 2014 CE. The colored circles represent ≥ category 2 hurricanes that passed within 50 km of TPBH in the instrumental record, where larger (smaller) circles represent more proximal (distal) passage, and color represent Saffir-Simpson intensity. Coarse sediment anomalies (CSA) are peaks that exceed the 11-point moving average (dashed black line). The purple dashed line is the 4.39% D_>63 µm_ CSA threshold, and CSAs that exceed this threshold are marked with purple triangles. (**b–e**) Hurricane events from the IBTrACS v4^1, 2^ within 115 km of TPBH from 1850 to 2019 CE in intervals from (**b**) 2019 to 1991 CE, (**c**) 1990 to 1951 CE, (**d**) 1950 to 1901 CE, and **e**) 1900 to 1850 CE. Color of the storm segment denotes its Saffir-Simpson intensity category at the time of measurement based on the subset USA_Agency_SSHS, which is derived from HURDAT_ATL maximum wind speed data*.* The grey circle represents a 115 km radius around TPBH. Optical satellite imagery was obtained from ESRI compiled satellite imagery. Maps were made using the USA Contiguous Equidistant Conic projected coordinate system^74^ to accurately preserved track distanced from TPBH.

First, as depicted in Supplementary Table S3, all hurricanes (category 1–5) that passed within 115 km of TPBH from 2014 to 1850 CE were considered for their potential to generate the youngest event beds (E1 to E11, Fig. 4a,b). Layer E1 was deposited between 2015 and 2005 CE (median age 2013 CE, E1 in Fig. 4a) and is likely attributable to Hurricane Irene (category 2), which passed ~ 15 km east of TPBH on 25 August 2011 CE or Hurricane Sandy, which passed ~ 45 km east of TPBH on 26 October 2012 CE. The only other hurricane that came within 115 km during this time was Hurricane Noel (category 1), which passed ~ 80 km east on 1 November 2007, but this event was likely too weak and distal to create a tempestite. The deposit E1 was most likely Irene based on its intensity and proximity, but Hurricane Sandy cannot be ruled out given the inability of the sediment records to differentiate events within the same calendar year.

Events E2-E4 were deposited between 2015 to 1972 CE (2σ range), and seven hurricanes passed within 115 km of TPBH from 2012 and 1992 CE (Fig. 4a,b). Assuming we have correctly attributed E1 to Hurricanes Irene and/or Sandy, deposit E2 (median age 2004 CE, 2σ age-range: 2011 and 1995 CE) is likely attributable to Hurricane Frances (category 2, ~ 40 km south on 24 September 2004 CE), or Hurricane Jeanne (category 3, ~ 50 km north of TPBH on 25 September 2004 CE). Hurricane Floyd also passed just 15 km to the east of TPBH on 14 September 1999 CE as category 3 hurricane, but it is more likely that Floyd deposited E3 (1995 CE median age, 2σ age-range: 1982 to 2004 CE). Category 1 Hurricane Dennis passed 60 km to the northeast on 28 August 1999, but was unlikely to deposit E3 because of its lower intensity and more distal passage in relation to Hurricane Floyd. Deposit E4 (median age 1986 CE) dates to between 1996 and 1972 CE, and only category 1 Hurricane Erin (22 km east; 1 August 1995 CE) and category 4 Hurricane Andrew (112 km south; 23–24 August 1992 CE) occur during this time. Given that E4 narrowly meets the 4.39% D_>63 µm_ CSA threshold, it is reasonable to interpret that it is related to a weaker proximal storm like Erin (~ 25 km south of TPBH) or a more distal but intense storm like Andrew (~ 112 km south of TPBH). However, Erin (1995 CE) is barely within the upper 2σ age-limit, so Hurricane Andrew is more likely responsible for E4.

Equally important is the absence of coarse sediment anomalies with median ages that fall between 1990 to 1951 CE when there is little regional hurricane activity (i.e., no false positives). No hurricanes passed within 50 km of TPBH, but four storms passed within 115 km (Fig. 4c): (i) Hurricane Betsy (younger, category 3) passed 70 km to the east on 6 September 1965 CE, (ii) Hurricane Janice (category 1) passed 100 km east on 7 October 1958 CE, (iii) Hurricane Betsy (older, category 3) passed 84 km to the east on 14 August 1956, and (iv) Hurricane Able (category 1) passed 90 km to the northeast on 18 May 1951 CE. None of these storms caused meaningful coarse sediment deposition. The lack of false positives from 1951 to 1990 CE increases confidence that coarse sediment anomalies in the TPBH composite record can be reliably attributed to hurricane passage through time.

Observational uncertainty in the IBTrACS dataset increases prior to 1970 CE because this precedes the satellite era^53^. This uncertainty further increases prior to aircraft monitoring around 1945 CE^53, 54^. Five coarse sediment anomalies were deposited at TPBH from 1901 to 1950 CE (E5-E9), when regional hurricane activity was elevated (6 hurricanes passed within 50 km and 14 passed within 150 km of TPBH). This includes a category 5 unnamed hurricane whose center of circulation passed just 4 km to the east in 1932 CE (Fig. 4a,d). The uppermost event bed deposited in the first half of the twentieth century is E5 with a median age of 1941 CE (2σ age-range: 1961 to 1915 CE, Fig. 4a). While this deposit could have been associated with one of the nine potential events between 1961 and 1915 CE, the most recent event after the ~ 40 year lull in activity between 1990 and 1950 CE is a category 3 hurricane that passed 43 km northeast of TPBH on 21 September 1947 CE. A category 1 hurricane passed 30 km to the southeast of TPBH on 12 September 1946 CE, so it is possible that both storms contributed to E5. Increasing uncertainty in track and intensity of hurricanes recorded prior to 1940 CE along with increasing age-uncertainty in the sedimentary age model limit confidence in the correlation between specific hurricanes and event beds. But, a qualitative assessment of this older time interval does reveal similar associations between event beds and hurricane magnitude as the post-1940 CE record SUPPLEMENTAL 4 (Supplementary Table S4).

In summary, 4 of the 5 of event beds with median ages between 1940 and 2014 CE can be confidently attributed to ≥ category 2 hurricanes that pass within 50 km of TPBH (Supplementary Table S3), with event bed E4 also most likely caused by a category ≥ 2 event. This encompasses all possible ≥ category 2 hurricanes that passed within 50 km of TPBH between 1940 and 2014 CE (not counting events occurring within the same or adjacent years, like Francis and Jeanne in 2004). While it cannot be ruled out that a proximal category 1 hurricane such as Erin (1995) could have deposited an event bed in TPBH, there is no deposit between 1850 and 2014 CE that is clearly attributable to this type of storm alone (Supplementary Table S3 and S4). This would suggest that TPBH records most ≥ category 2 hurricanes that pass within a 50 km radius since 1940 CE, and may additionally archive more intense (category 4–5) distal hurricanes (> 50 km away from TPBH). As such, coarse event beds older than 1940 CE are likely emplaced by hurricanes with similar characteristics.

### Northern Bahamas hurricane frequency

The new high-resolution record from TPBH revises and considerably expands upon previous records^3, 24^ of hurricane activity from the northern Bahamas. The previously published age-model for TPBH-C1 showed a dramatic reduction in sedimentation rate at 1950 CE from 1.6 cm yr^-1^ to 0.1 cm yr^-1^. The new composite TPBH record has a mean sedimentation rate of ~ 1.3 cm yr^-1^ spanning the complete 888 cm record, and the event beds can be visually correlated between TPBH-C1 (2014 core sample) and the upper 164 cm of the new TPBH composite record (e.g., peak at 12, 24, 100 cm in TPBH-C1 to 15, 35, and 109 cm in TPBH composite record; Fig. 3). This suggests that the previous TPBH-C1 age-model is skewed too old prior to 1950 CE. The age-model for the TPBH composite record was built largely from terrestrial plant macrofossils from two new cores with correlative stratigraphy (TPBH-C2 and TPBH-C3, Fig. 3). The terrestrial plant macrofossil dates are less likely to be influenced by the marine carbon reservoir, and therefore more reliable than the radiocarbon dated *B. domingensis* bivalves from TPBH-C1. Given the increased confidence in the new TPBH composite record age-model, the original 164 cm stratigraphy sampled by TPBH-C1 actually represents only a continuous ~ 100 year record (~ 1910 to 2011 CE), in contrast to the ~ 1000 years originally reported^24^. This means, that while the original TPBH-C1 reconstruction does suggest that hurricane activity increased between 1350 and 1650 CE, any similarity with the new TPBH composite record is entirely coincidence. As such, only the new TPBH composite record should be used when assessing hurricane activity at TPBH prior to 1910 CE.

**Figure 5:**
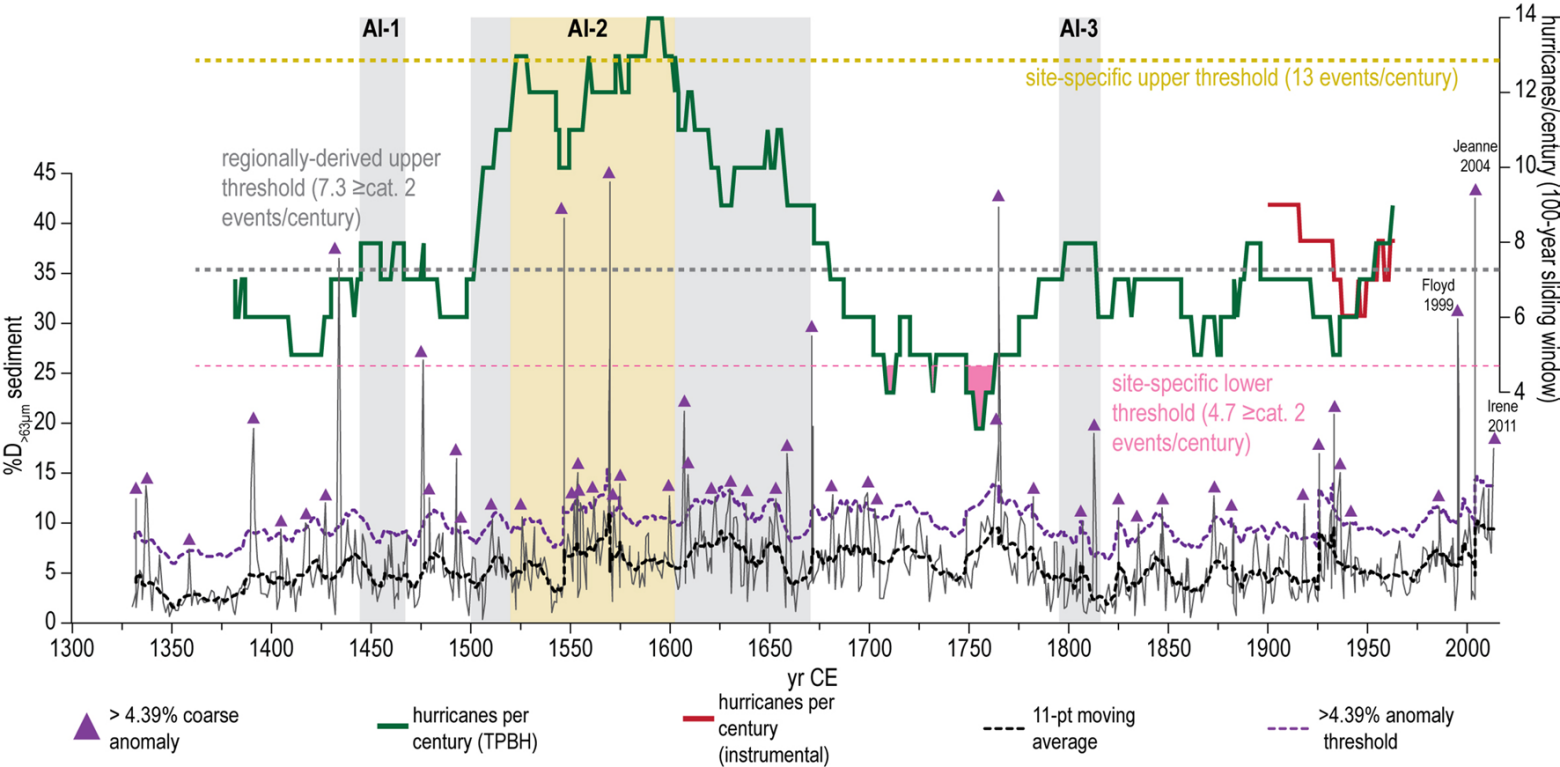
Hurricane events per century recorded in Thatchpoint Blue Hole from 1330 to 2014 CE. Coarse sediment (%D_>63 µm_) plot (grey line) from TPBH composite record from 1330 to 2014 CE. Coarse sediment anomalies (CSA) are peaks that exceed the 11-point moving average (dashed black line). The purple dashed line is the 4.39% D_>63 µm_ CSA threshold, and CSAs that exceed this threshold are marked with purple triangles. The red line represents 100-year sliding window counts for events based on the IBTrACS v4 observational data^1, 2^, and the green line represents 100-year sliding window counts for significant peaks in the composite TPBH record. Event frequency significance thresholds are depicted as: gold dashed line is the site-specific upper 90th percentile threshold of 13 ≥ category 2 events per century, grey dashed line is the regionally derived upper 90th percentile threshold of 7.3 ≥ category 2 events per century, thin light-pink dashed line is the site-specific lower 10th percentile threshold of 4.7 ≥ category 2 events per century, thin red-pink dashed line is the regionally derived lower 10th percentile threshold of 1.6 ≥ category 2 events per century. The temporal windows where events per century exceed/fall-below these upper/lower thresholds are filled in with boxes that correspond to the color of the threshold lines.

**Figure 6:**
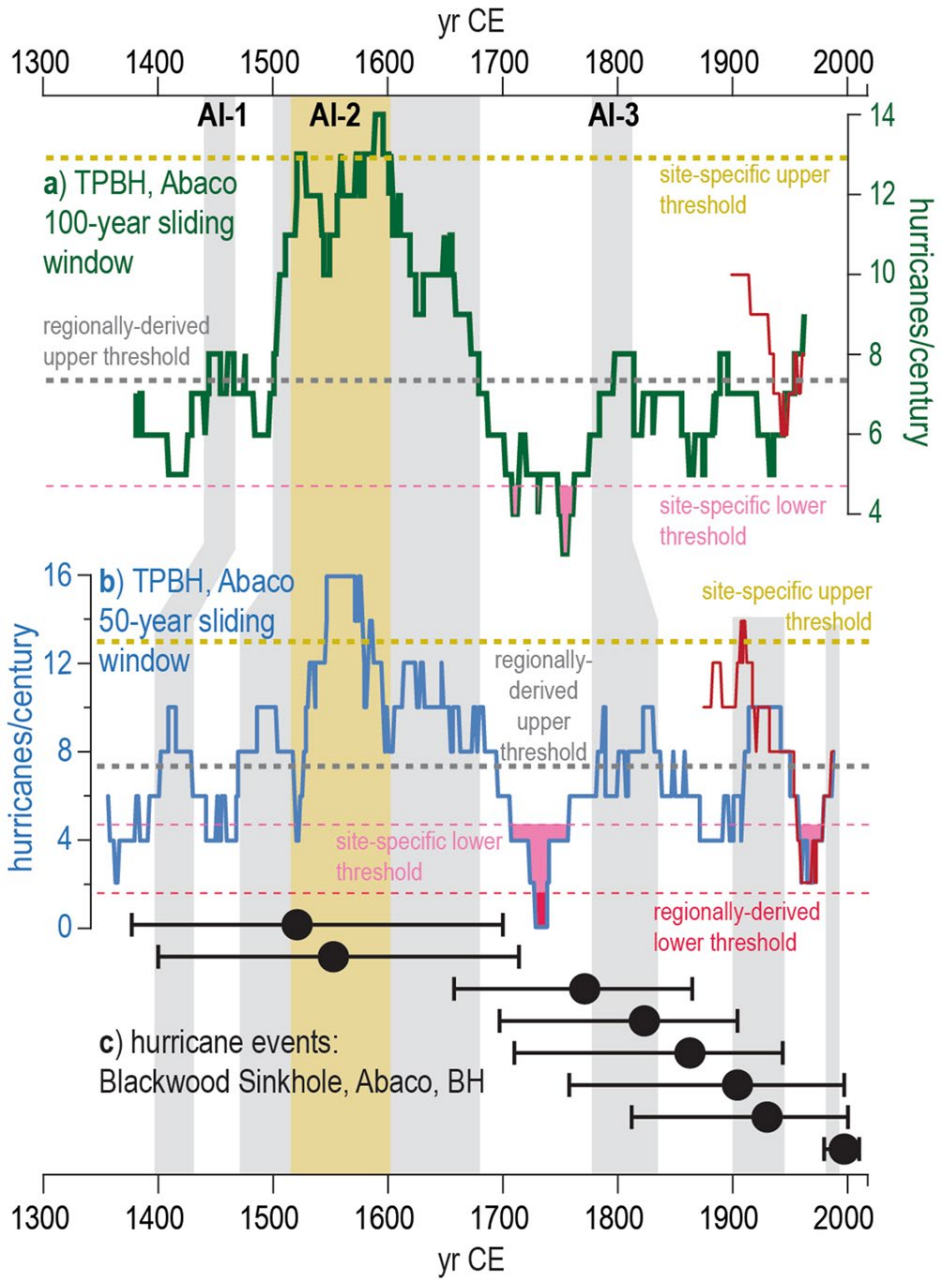
Comparison of paleo-hurricane records from Abaco. (**a**) The solid red line represents 100-year sliding window counts for events based on IBTrACS v4 data^1, 2^, and the green line represents 100-year moving window counts for significant peaks in the composite TPBH record. (**b**) The red line represents 50-year moving window counts for events based on IBTrACS v4 data, and the blue line represents events per century calculated from 50-year sliding window counts for significant peaks in the composite TPBH record. In (**a**) and (**b**), event frequency significance thresholds are depicted as: gold dashed line is the site-specific upper 90th percentile threshold of 13 ≥ category 2 events per century, grey dashed line is the regionally derived upper 90th percentile threshold of 7.3 ≥ category 2 events per century, thin light-pink dashed line is the site-specific lower 10th percentile threshold of 4.7 ≥ category 2 events per century, thin red-pink dashed line is the regionally derived lower 10th percentile threshold of 1.6 ≥ category 2 events per century. The temporal windows where events per century exceed/fall-below these upper/lower thresholds are filled in with boxes that correspond to the color of the threshold lines. (**c**) Black circles represent the mean age of hurricane events recorded in Blackwood Sinkhole in northern Abaco^3^ and the associated 2σ-age uncertainty is marked by the black lines extending from each circle.

**Figure 7:**
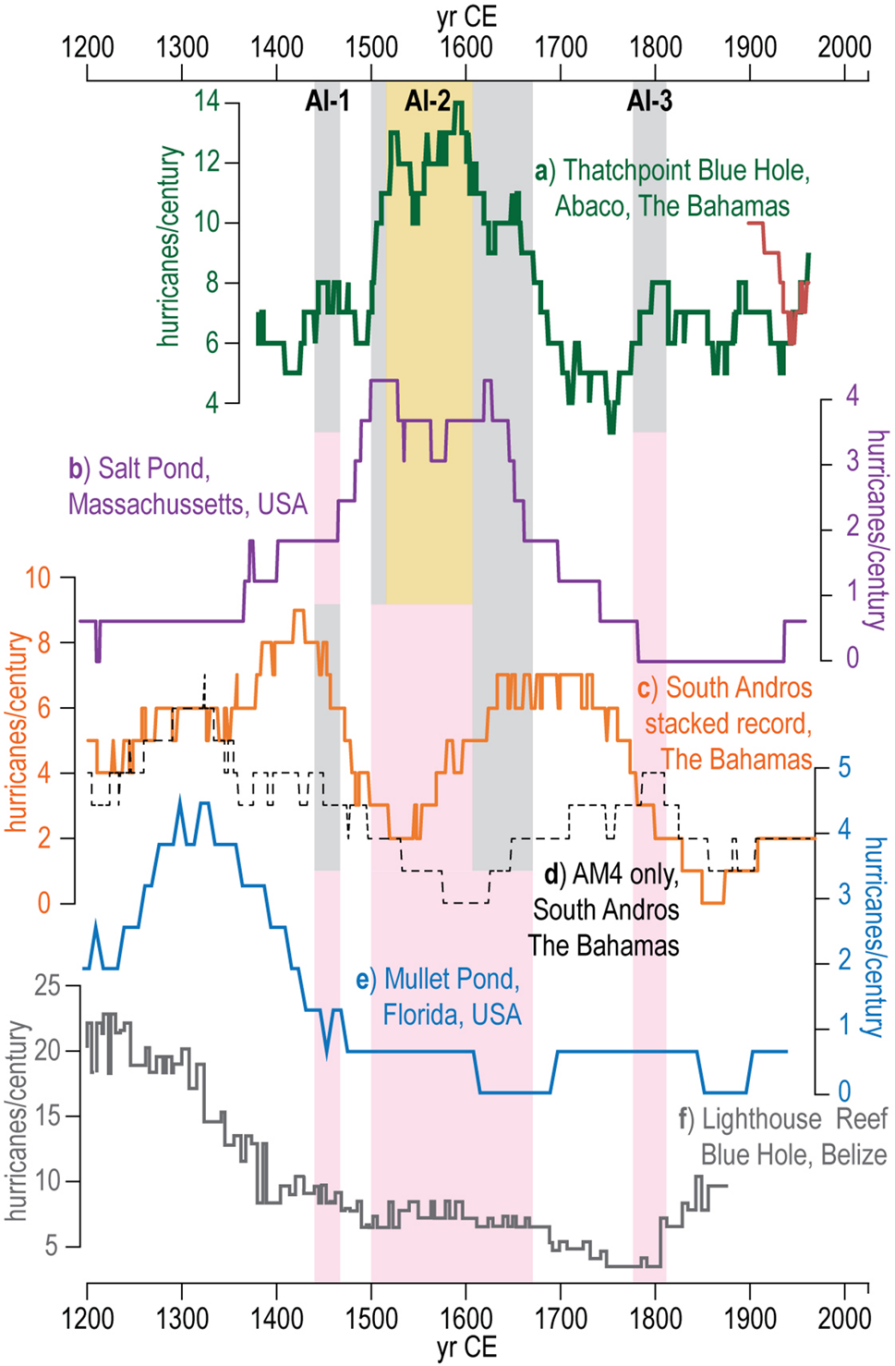
Comparison of high-resolution paleo-hurricane records from throughout the North Atlantic. Hurricane events per century based on sedimentary reconstructions of regional hurricane frequency per century over the last 800 years at sites throughout the North Atlantic including: (**a**) TPBH 100-year sliding window counts (green line), (**b)** Salt Pond in Massachusetts^18^ (purple line), (**c**) South Andros, The Bahamas stacked record presented in this study but derived from Wallace, et al.^25^ (orange line) and (**d**) AM4 (single record) in South Andros^25^, (orange line). (**e**) Mullet Pond on the Florida Gulf of Mexico Coast^27^ (blue line); (**f**) Lighthouse Blue Hole, Belize^23^ (grey line). The gold and grey boxes correspond to TPBH Composite record 100-year count statistical active intervals as defined in Fig. 5. The pink boxes match the temporal extent of these active intervals, but indicate lack of correlation between the timing/magnitude in the other records.

We define active intervals (AIs) using both a regionally-derived (grey boxes in Figs. 5, 6, 7) and site-specific (gold boxes in Figs. 5, 6, 7) upper-level significance threshold of 7.3 and 12.4 ≥ category 2 events per century generally within 50 km, respectively (see METHODS section Determining Active Hurricane Periods). The most prolonged active period at TPBH occurs between 1500 to 1670 CE (AI-2), with a mean count of 11 hurricanes per century (AI-2, Fig. 5). Based on the regionally-derived upper threshold (7.34 ≥ category 2 events per century), there is a 90% statistical likelihood that stochastic forcing alone cannot explain the elevated hurricane frequency from 1500 to 1670 CE. Furthermore, the period from 1520 to 1600 CE also exceeds the site-specific upper threshold of 13 ≥ category 2 events per century, with an up to 14 events per century (average of 12.3 events per century, *n* = 82).

Two other brief periods from ~ 1440 to 1470 CE exceed the regionally-derived upper 90% confidence interval of 7.3 ≥ category 2 events per century: (i) AI-1, average 7.7 events per century), and (ii) AI-3 from ~ 1795 to 1815 CE (average of 8 events per century), but these intervals do not reach activity levels that exceed the site specific upper threshold. It should be noted that the red line in Figs. 5 and 6 indicate the number of hurricane events within 50 km between 1850 and 2014 CE expected to be detected by TPBH based on the sensitivity analysis, which means that deposits from Hurricanes Frances in 2004 (category 2) and an unnamed Hurricane in 1933 (category 3) were not counted as they occurred in the same or subsequent year as a more powerful or proximal hurricane. If these storms are included, the frequency counts throughout most of the last 164 years exceed the upper limits of the regionally derived threshold with an average of 13.2 events per century (*n* = 64 100-year windows).

These centennial-scale active intervals are also apparent at a multi-decadal scale (50-year sliding window counts, Fig. 6), short lived (multi-decadal) extremes in hurricane frequency are more pronounced when events per century is calculated based on 50 year sliding window counts. In the 50-year sliding window counts, AI-1 spans from 1400 to 1425 CE. When considering the regionally derived upper threshold of (7.3 ≥ cat. 2 events per century within 50 km), the length of AI-2 is extended to initiate around 1475 CE and end around 1700 CE. During the peak of AI-2 (1520 to 1600 CE), TPBH records up to 16 distinct event deposits per century (50-year sliding window) from 1547 to 1576 CE. The duration of AI-3 is extended by ~ 30 years, occurring from ~ 1780 to 1850 CE. Substantially lower hurricane frequency from 1700 to 1760 CE and 1960 to 1980 CE is emphasized in the 50-year sliding window counts, although events frequency per century briefly dips below the site-specific lower threshold (4.7 ≥ cat. 2 events per century) in the 100-year sliding window counts during the 1700 to 1760 CE interval (Fig. 6). Two additional periods from 1880 to 1940 CE and 1990 CE to present exceed the regional activity threshold in the 50-year sliding window counts, that are not significant in the 100-year sliding window counts. However, given that average 2σ age-uncertainty throughout the TPBH composite record is ± 24.4 years at each 1 cm interval, it is not clear if brief periods of enhanced activity that are only significant at the 50-year sliding window scale are driven by shorter lived (multi-decadal) external forcing (i.e., climate) or simply represent stochastic variability in hurricane activity.

A lower-resolution hurricane record (both: increased age uncertainty and lower capacity to observe storm passage) is available from Blackwood Sinkhole^3^, which is positioned 60 km northeast of TPBH on the eastern shoreline of Abaco. Blackwood Sinkhole only archived two intense hurricane events (≥ category 3 events) during the active interval from 1500 to 1675 CE (AI-2), but the age uncertainties for these events are large. In contrast to TPBH, Blackwood Sinkhole preserves the most event beds between 1790 and 1940 CE (five events recorded in Blackwood Sinkhole, Fig. 6), a period when TPBH records 12 events based only on the median age of coarse sediment anomalies, and up to 20 events during the maximum 1650 to 2000 2σ age-uncertainty for Blackwood Sinkhole during this period. Despite recording substantially more events than Blackwood Sinkhole record, there is no time between 1790 and 1940 CE in which the TPBH composite record centennial-scale event counts exceed the site-specific or regional statistical upper thresholds. TPBH centennial scale AI-3 (~ 1795 to 1815 CE) coincides with three events recorded in Blackwood Sinkhole (1771 CE ± 103 years, 1822 ± 134 years, and 1860 ± 116 years). Perhaps most interesting is that Blackwood Sinkhole archives no events beds with mean ages between ~ 1700 to 1780 CE when the composite record from TPBH has the fewest events per century (average of 4.8 events per century, *n* = 76, three recorded events in 76 years, Figs. 5, 6).

It should be cautioned that one should not infer changes in storm *intensity* in the last 700 years based on the relative sensitivity of Blackwood Sinkhole (i.e., less sensitive) versus TPBH (i.e., more sensitive) to hurricane-induced sediment deposition. For example, it could be tempting to assume more intense hurricane strikes on Abaco based on more frequent events beds during the last 200 years in Blackwood Sinkhole (Fig. 6). However, Blackwood Sinkhole has an average 2σ age-uncertainty of ± 110 years at each 0.5 cm interval from 1300 to 2013 CE, and a much lower overall sedimentation rate for the entirety of the record (0.3 to 0.6 mm yr^-1^, last 3000 years) when compared to TPBH (~ 1.3 cm yr^-1^, last 700 years). These differences in sedimentation rate and time averaging mean that TPBH records 53 events and Blackwood Sinkhole records 6 events during the same ~ 700-year span. The lower sedimentation rate, increased timing uncertainties, and fewer storm deposits during the instrumental period also mean that the types of hurricanes that produce favorable conditions for event bed deposition into Blackwood Sinkhole is less understood.

Sedimentary processes also significantly differ between each site: sedimentation in Blackwood Sinkhole on the terrestrial landscape versus TPBH in a subtidal marine setting with locally abundant sediment production and supply. The coarse-grained events beds in Blackwood Sinkhole comprises limestone fragments and brackish invertebrates, which are attributed to hurricane mobilization of particles from the adjacent terrestrial surface, wetlands, and vertical aquatic habitat on the sinkhole walls^3, 55^. This means that the Blackwood Sinkhole hurricane signal is likely also linked to hurricane-induced flooding (surge-induced effects on the local groundwater table and rainfall). In contrast, TPBH in a subtidal lagoon setting is generating events beds from subaqueous sediment redistribution during elevated storm surge and local hydrodynamics during a hurricane event^36, 56^. In balance, the Blackwood Sinkhole record remains valuable because it extends deeper in time than the TPBH composite record (~ 2300 years longer, 3000 cal yrs BP total), the sedimentation rate is continuous with no hiatuses during the last 3000 years, and does provide a higher-resolution replication of evidence for intense hurricane activity at Puerto Rico^19^. However, when considering the last 700 years, the dramatically increased sedimentary resolution and reduce temporal uncertainties means the composite record from TPBH vastly improves our understanding of hurricane activity in the northern Bahamas.

### Comparison to North Atlantic paleo-hurricane reconstructions

The northern Bahamas has experienced some of the highest levels of ≥ category 2 hurricane strikes within 50 km since 1850 CE (11–16 events) in the North Atlantic Ocean, along with the Gulf coast region (offshore Mississippi delta), northwestern Cuba, St. Johns and Puerto Rico, and just offshore of the Carolina Outer Banks (Fig. 1). In contrast, other areas experience much lower activity (3–4 ≥ category 2 hurricane strikes since within 50 km since 1850 CE), such as a lane between northern Cuba and Andros Island in the central Bahamas, the southern Gulf of Mexico, the southern Yucatan Peninsula, the northern coasts of Haiti and The Dominican Republic, the northern Gulf Coast of Florida, Jamaica, and the southern coast of Cuba (Fig. 1). It is likely that geography plays a role in this spatial variability by creating a “shadow effect” that is observable behind larger landmasses in Fig. 1, wherein storms weaken or dissipate while passing over land. For example, lower activity in the southern Gulf of Mexico likely results from hurricanes losing strength while passing over the Yucatan Peninsula. Similarly, Cuba likely shields the area south of Andros Island (The Bahamas) from many hurricanes that approach from the southern Caribbean.

High-resolution (near annual-scale) paleo-hurricane reconstructions can help assess if these recent spatial geographic relationships of hurricane activity persisted through prehistory. Indeed, increased hurricane strikes in the northern Bahamas between 1500 to 1670 CE is synchronous with evidence for elevated hurricane activity at higher latitudes on the U.S. eastern seaboard. For example, up to four ≥ category 2 hurricane strikes per century occurred at Salt Pond in Massachusetts from 1525 to 1600 CE^18^, and increased hurricane event beds are preserved in subsurface sediments in Mattapoisett Marsh in Massachusetts from 1400 to 1650 CE^57^ (Fig. 7b). Increased barrier island inlet formation increased on the Outer Banks of North Carolina^58^ and enhanced salt marsh erosion in Connecticut^59^ between 1400 and 1600 CE also suggest more frequent hurricane strikes along the U.S. Eastern Seaboard during this time. Colonial-era shipwrecks increased during this interval^60^, including 17 wrecked ships off Abaco during one event in 1595 CE^61^ that could be archived as one of three event beds in TPBH whose age-uncertainties overlap this hurricane. A historical reconstruction of tropical cyclone activity based on Spanish historical documents from the Archivo General de Indias^62^ provides further evidence of more hurricanes impacting naval activity during this time. Additionally, suppressed pine growth (based on tree ring data) from Florida indicates heightened activity between 1500 and 1650 CE^60^.

In contrast, the hurricane passage history in the northern Bahamas and eastern North America is different from the central Bahamas. For example, the near-annually resolved Common Era record of hurricane activity on South Andros^25^ (METHODS section describes compiling paleo-records from 3 different blue holes into a single South Andros stacked record, Fig. 7c,d) is largely anti-phased with the composite TPBH record over the last 684 years, yet these sites are only separated by ~ 280 km (northeast to southwest, Fig. 7d). Given that these sites document generally similar hurricane types (≥ category 2–3 passing within 50 km) and have similar sedimentation rates and age-uncertainties (1.3 cm yr^-1^ with ± 24.4 yr 2σ uncertainty at each 1 cm interval at TPBH, ~ 1–1.4 cm yr^-1^ ± 38 yr 2σ uncertainty in South Andros records), they present ideal case studies to assess long-term patterns of regional hurricane frequency (Fig. 1). When TPBH documents up to 14 events per century (mean 12.3 events per century, *n* = 82), the South Andros stack documents only 2 to 5 events per century (mean 3.2 events per century, *n* = 82). Throughout this entire active interval from ~ 1500 to 1670 CE when TPBH experiences an average 11 hurricane strikes per century (*n* = 178), the South Andros stack only documents a mean 4.3 events per century (*n* = 170, Fig. 7). In contrast, when the South Andros stack documents 6 events per century between 1700 to 1775 CE, TPBH experiences the lowest activity observed over the last the 684 years (mean 4.8 events per century *n* = 76). It is also worth noting that TPBH (mean 7.7 events per century from 1330 to 2014 CE, *n* = 583 intervals) records more events overall than South Andros during the same time frame (average of 4.4 events per century, *n* = 590 intervals), which is consistent with spatial heterogeneity in regional storm frequencies during the instrumental period (Fig. 1).

At a broader geographic scale, the timing for enhanced paleo-hurricane activity in The Bahamas differs from paleo-hurricane records in the northeastern Gulf of Mexico and Yucatan Peninsula over the last millennium. Elevated activity recorded by TPBH from 1500 to 1670 CE (AI-2) corresponds with decreased activity both in the Gulf coast region of Florida^27, 29^ and the eastern Yucatan Peninsula^23^. This interval documents a time in the past in which two sites that are relatively inactive during the observational record (3 to 4 ≥ category 2 events within 115 km since 1850 CE, Fig. 1) remain less active (Fig. 7). However, annual-scale hurricane reconstructions from these areas document that they have been much more active at times during the past, such as when Mullet Pond^27^ and Spring Creek^29^ (Florida) and Lighthouse Reef Blue Hole^23^ (Belize) document more frequent hurricane strikes from ~ 1100 to 1400 CE (Fig. 7e,f).

Confidence is emerging in evidence for regional and basin-wide paleo-hurricane patterns based on the growing array of high-resolution paleo-hurricane studies. It is well established that climate impacts hurricane genesis, track, intensification, and overall Atlantic activity (see comprehensive reviews in^4, 63–65^). However, stochastic processes may partly explain spatiotemporal changes in paleo-hurricane activity. This means that the recorded rate of hurricane landfalls over time at a given site can be driven by chance alone, and thus potentially mask an otherwise clear response to climatic forcing. Despite the potential for a stochastic component of the observed spatial–temporal patterns of paleo-hurricane activity, it should be noted that only 4 ≥ category 2 hurricanes passed within 50 km of South Andros between 1850 and 2019 CE compared to 14 such events in the northern Bahamas (Fig. 1). This would suggest that the spatial variability in the frequency of intense hurricane strikes over the last 169 years has persisted in some capacity throughout the last ~ 700 years. The high density of blue holes in the tropical North Atlantic Ocean, including on nearby Grand Bahama Island, provide promise for evaluating the results from TPBH both on the Little Bahama Bank and elsewhere.

### Conclusions and implications

The northern Bahamas (Abaco and Grand Bahama) have frequently experienced ≥ category 2 hurricane landfalls since 1850 CE, relative to most other places in the North Atlantic. Over the last ~ 700 years, however, a near-annually resolved record of prehistoric hurricane passage from a blue hole on Abaco Island, Thatchpoint Blue Hole (TPBH), indicates that the northern Bahamas has experienced periods with statistically-significant *higher* frequencies of hurricane passage. From 1900 to 2014 CE, eight ≥ category 2 hurricanes passed within 50 km of TPBH, and an additional five ≥ category 4 events passed within 115 km. From 1900 to 2014 CE, the TPBH composite sediment record archived nine coarse sediment event beds within age-model uncertainties for passage of these hurricanes, providing unprecedented insight into the types of hurricane events that are most likely to cause event bed deposition in TPBH. Most ≥ category 2 events within 50 km and ≥ category 4 events within 115 km appear to be recorded within TPBH. This new record revises a previous study^24^ and demonstrates a nearly linear sedimentation rate at this blue hole of 1.3 cm yr^-1^ spanning the last ~ 700 years. Most importantly, the period from 1500 to 1670 CE was characterized by especially high levels of hurricane activity in the Northern Bahamas, similar to the eastern seaboard of North America. At TPBH, up to 14 ≥ category 2 hurricane strikes occurred per century, which represents a minimum estimate of the number of regional hurricane events. This active period is unlikely to be the result of stochastic variability alone because event counts exceed the upper 90th percentile cutoff threshold based on calculated Poisson probability.

This study provides further evidence for spatial heterogeneity in hurricane strike frequency across the North Atlantic Basin over the last 700 years, which is apparent in observational data since 1850 CE. For example, while the northern Bahamas (TPBH) experienced enhanced hurricane passage from 1500 to 1670 CE, this is a period of relative quiescence in the Gulf of Mexico, central Bahamas (South Andros), and southern Caribbean (Belize). Spatial heterogeneity in paleo-hurricane activity is further apparent when comparing the record of hurricane passage at TPBH to South Andros, where hurricane frequency is generally antiphased over the last ~ 700 years, despite being only ~ 280 km apart. This could suggest that The Bahamas is something of a crossroads for hurricane activity. Given this spatial heterogeneity, it is critical that (i) we continue to produce high-resolution paleo-hurricane records to better constrain the potential for multidecadal and centennial variations in Common Era hurricane activity, and (ii) that multiple sites from the same region are compiled and examined holistically before meaningful relationships between climate and hurricane activity can be fruitfully established in the broader areas of the north Atlantic. Nevertheless, multi-centennial regionally coherent patterns in hurricane activity are beginning to emerge across the Atlantic, such as the well replicated active interval along the North American Eastern Seaboard from 1450 to 1650 CE.

## Methods

### Sediment collection and analysis

Sediment vibracores (7.5 cm diameter) were collected in January 2015 (prior to Hurricane Dorian in 2019) by deploying a Rossfelder P3 submersible vibracoring system from the surface on a portable raft that was positioned in the center of TPBH. The new cores collectively sampled the upper 888 cm of stratigraphy. The collected sediment–water interface was preserved in TPBH-C2 (615 cm; 26.3234° N, 77.2934° W) and TPBH-C5 (194 cm; 26.3233° N, 77.2934° W), but the Rossfelder P3 over-penetrated when collecting TPBH-C3 (836 cm, 26.32339° N, 77.29342° W) so the sediment–water interface was lost. TPBH-C3 and TPBH-C5 were taken within 1 m of each other, with TPBH-C2 taken within 5 m of these cores (Supplementary Fig. S2). The continuous sediment vibracores were sectioned into lengths up to 150 cm for transport back to the laboratory. During the sectioning of TPBH-C3, rapid sediment expansion caused by depressurizing hydrogen sulfide gas caused the loss of sediment from 410–447 cm core depth at a section break. A final composite and complete 888 cm stratigraphic record from TPBH was compiled by combining the preserved sediment–water interface from the upper 16 cm from TPBH-C5, the entirety of the longest core (TPBH-C3), and the stratigraphically-equivalent interval from TPBH-C2 (426–463 cm, established through correlative stratigraphy) that was lost during sectioning of TPBH-C3 (410–447 cm).

In the laboratory, all sediment core sections (TPBH-C2, TPBH-C3, TPBH-C5) were split lengthwise for photography, x-radiography, qualitative sediment description and quantitative textural analysis. Using similar methods to van Hengstum, et al.^24^, sediment textural variability was analyzed in all cores using a standard loss on ignition procedure (LOI) combined with manual sieving over a 63 μm mesh (Fig. 3). Contiguous sediment subsamples (~ 2.5 cm^3^) were obtained at 1 cm intervals downcore, desiccated overnight at 80 °C, and then ignited at 550 °C to re-mineralize organic carbon^66^. The remaining carbonate sediment residue for each sample was manually wet sieved on a 63 µm mesh, re-desiccated at 80 °C, and the reweighed to determine % mass (mg) of coarse particles > 63 µm per total sampled mass of sediment (%D_>63 µm_ ) content in each sample (Fig. 3).

### Age control

To independently constrain the upper portion of the TPBH record (top 510 cm) and the observational period in our record (1850–2015 CE) in particular, seven samples were analyzed downcore at 70–80 cm intervals (*n* = 7 from 1620 to 2015 CE) to identify any palynological evidence of colonial disturbance on the adjacent landscape from permanent settlements that were established as early as 1783 CE^67^. Pollen was processed following the methodology of Agosta G’meiner^68^, and counted to over 100 total grains per sample for all but one of the seven samples (SUPPLEMENTAL 3 subsection Opaque Spherules and Pollen, Supplementary Table S2). Total fossil pollen concentrations ranged from 16,200 to 5,657 grains/cm^3^ (Supplementary Table S2). The most substantial observation was a peak in opaque spherules at 140 cm, which are likely derived from the combustion of fossil fuels. Due east of TPBH, there was a short-lived settlement from 1906 to 1916 CE called “Wilson City” associated with the early twentieth century Abaco pine logging industry^69^. This settlement included a rail system, logging town, and 300 ft. pier servicing ships that likely utilized coal. Therefore, 140 cm was assigned an age of 1911 ± 5 years in the sedimentary age-model. The stratigraphic position of the peak is consistent with the previous radioisotopic evidence from TPBH-C1 that the upper ~ 170 cm of sediment dates to the within the last 100 years. ^137^Cs activity was measured downcore in the top 140 cm of the core in 5–10 cm interval to constrain the chronohorizon associated with the onset and moratorium of nuclear weapons testing in 1954 CE and the 1963 CE, respectively. The results of this analysis were used as an independent test of the veracity of the sedimentary age-model for the TPBH composite record, and the precise methods used for this analysis are discussed in SUPPLEMENTAL 3 subsection Gamma Dating: Methods and Results.

To develop age control for the older part of the record, available plant remains (e.g., mangrove leaves and unidentifiable organic matter) and two basal gastropods were submitted for radiocarbon dating from TPBH-C3 (*n* = 16) and TPBH-C2 (*n* = 2 dates, Supplementary Table S1). As previously mentioned, no *Barbatia domingensis* bivalves were found in any of the new cores extracted from the center of TPBH, and terrestrial plant macrofossils were rare. Samples submitted for radiocarbon dating did not originate from a stratigraphic level that was classified as a coarse sediment anomaly (see METHODS subsection Event Threshold Attribution and Frequency), which helps limit the possibility of radiocarbon dating older material that was reworked during a high-energy event. The dates submitted from both TPBH-C3 and TPBH-C2 provided an independent assessment of sedimentation rate in two separate cores from TPBH, but all dates were subsequently used in developing the final downcore age-model for the 888 cm composite record.

Conventional radiocarbon results were calibrated into years before present (cal yrs BP) with consideration for the source of carbon incorporated into the analyzed sample (i.e., marine vs. terrestrial reservoirs), and the fraction modern value (F^14^C exceeding 1.0 post-date 1950 CE). One mangrove leaf from 61.5 cm in TPBH-C3 was calibrated with the Northern Hemisphere Zone 2 dataset (NHZ2) in CALIbomb because its fraction modern carbon value (F^14^C) exceeded 1.0000^70^. Organic remains with a stable carbon isotopic value (δ^13^C_org_) value more depleted than − 18.0‰ were calibrated with IntCal13. However, the organic remains with more enriched δ^13^C_org_ values of − 17.0‰ and − 8.8‰ were deemed as likely marine sourced organic remains (e.g., sea grass fragments), and the conventional radiocarbon results on these samples were subsequently calibrated using Marine13 ^43^. Finally, two radiocarbon dates at the base of TPBH-C3 on intertidal gastropods (*Cerethium sp.*) were calibrated with a mixed 50/50% calibration curve to reflect their likely incorporation of both marine- and terrestrially-derived carbon into the production of their biogenic carbonate.

A final age-model with 95% confidence intervals for the 888 cm composite TPBH record was developed using Bayesian statistical approaches in the R package Bacon v2.2^71^ (Fig. 3). The final 15 control points used in the age-model were a core-top age of 2015 CE, the fossil fuel combustion spherule peak at 140 cm dated to 1906 to 1916 CE, and 13 radiocarbon dates (*n* = 11 from TPBH-C3, *n* = 2 from TPBH-C3). Dates from both TPBH-C3 and TPBH-C2 were included in the age-model for the composite 888 cm sedimentary record, with the depths in the composite record assigned to the dates from TPBH-C2 based on correlative stratigraphy. Coarse grained sedimentary layers that were thicker than 5 cm were assumed to have been deposited instantaneously, based on their likely relationship to hurricane passage, so these coarse layers were treated as geologically instantaneous in the downcore age-model (indicated by orange bars in Fig. 3).

### Event threshold attribution and frequency

The methods of to identify the most likely intervals (i.e., event beds) where coarse-grained sedimentation substantially exceeds background sedimentary conditions were modified from Donnelly, et al.^18^, Wallace, et al.^25^, Lane et al.^27^. First, an 11-point moving average of data excluding those with a coarse fraction value > 12% (upper 90th percentile of %D_>63 µm_) was subtracted from all %D_>63 µm_ data to establish a series of coarse sediment anomalies (CSA). Deposits were classified as hurricane events if the CSA exceeded the upper 90th percentile of the CSA cumulative distribution (CSA = 4.39% D_>63 µm_). The suitability of this threshold was assessed by comparing the timing and number of deposits that exceed this threshold to the number of hurricanes that could have impacted TPBH during the last 164 years (1850–2014) based on the IBTrACS version 4 dataset that compiles observational records of hurricane activity since 1850 CE^1, 2^ (Discussion subsection Historical hurricane strikes: calibrating the record). Historical hurricanes were attributed to event beds by first determining the known hurricanes in the 2σ age-range of each event bed passed within or 50 and 115 km proximity thresholds. Starting with the most recent event bed (E1), we selected which hurricane(s) were most likely to leave a deposit in TPBH based on (i) how the date of the hurricane compared to the median age of the event deposit, (ii) intensity and proximity of the hurricane to TPBH, (iii) which quadrant of the hurricane impacted the area around TPBH the most, and (iv) translational velocity of the hurricane (slower events are more likely to leave a deposit). Once a hurricane was attributed to an event deposit, this hurricane and any others that occurred more recently than that event were ruled out for attributions below. Similar to Lane et al.^27^, hurricane event beds per century were counted using a 100-year sliding window to assess centennial-scale hurricane variability, as well as a 50 year sliding window from which events per 50-years counts were multiplied by 2 to normalize units to events per century (Figs. 5, 6). The 50-year window counts emphasize shorter (multidecadal) periods of extremely high or low hurricane activity.

### Determining active hurricane periods

Independent of the climate system, simple stochastic variation in cyclogenesis and storm tracks can naturally create time intervals where hurricane activity at a given locale may be greater or less than average conditions. This random variability must be considered when assessing any potential periods of active hurricane activity at a given locality. Assuming hurricane frequency at a given location follows a Poisson process, we calculate cumulative Poisson probability (*P*) of hurricane frequency at value (*k*) based on expected frequency (*λ*) as follows in Eq. (1):

1 $$P\left(k,\lambda \right)=1-{\sum }_{k=0}^{k}\frac{{\lambda }^{k}{e}^{-\lambda }}{k!}$$

Values for expected hurricane event frequency (*λ*) were based on a regional estimate calculated by Wallace, et al.^25^ (regional bounding 550 km radius circle centered over 24.08°N and 75.39°W). The expected regional frequency value (*λ*) of ≥ category 2 hurricanes passing within 50 km of any given point is 3.7 events per century, resulting in upper 90th percentile cutoff threshold of 7.3 events per century and a lower 10th percentile cutoff threshold of 1.6 events per century. To calculate site-specific regional threshold, we examine the event frequency from 1900 to 2014 CE rather than the entire observational record back to 1850 CE due to increased uncertainty regarding storm intensity and track location in these older records. It was determined that there were 12 hurricane events that were likely to have induced coarse-sediment deposition in TPBH from 1900 to 2014 CE (see DISCUSSION subsection: Historical Hurricane Strikes: Calibrating the Record for justification, Fig. 4). Of these twelve events, only nine coarse beds would be distinct in TPBH stratigraphy given the temporal resolution of this record (i.e., not counting multiple events within the same calendar year). This implies an average of 7.9 events per century which would result in an upper 90th percentile threshold of 13 events per century and a lower 10th percentile threshold of 4.7 events per century (Figs. 5, 6). As with the regionally derived thresholds, we calculated upper cutoff thresholds following the methods described by Ulm^72^. If the number of recorded storms per century exceeds (falls below) the upper (lower) threshold, there is a 90% probability that this increase (decrease) exceeds the influence of random variability and can be considered a period of active (quiescent) hurricane activity at a given locale.

### Compilation of South Andros paleo-hurricane records

The South Andros stack compiles the three different blue hole sediment records (AM2, AM5, and AM4) presented in Wallace et al.^25^. These data are accessible online at the National Climatic Data Center (https://www.ncdc.noaa.gov/dataaccess/paleoclimatology‐data). Wallace and others noted that these three different sediment records captured similar patterns of hurricane event frequency on South Andros while not capturing the exact same events. All three blue holes on South Andros were close enough to capture some, but not all, of the same events. Thus, when compiling these three records together, we sought to remove repeated representation of the same events among the three contributing records. To accomplish this, multiple events falling within the one sigma age-model uncertainties of each other were combined to represent a single landfall event on the island. We defined the date and one sigma age ranges for the combined events as the average of the dates and age ranges of the contributing events. When there was more than one event in a blue hole record falling within the age uncertainties of an event from another blue hole record, we consolidated the event in the first blue hole site that was closest in age to that captured in the second site. The main difference between the new South Andros stack presented here and the solitary paleo record from blue hole AM4 featured by Wallace et al.^25^ is a period of increased activity in the stacked record from ~ 1600 to 1780 CE.

## Supplementary information

Supplementary information

## Data Availability

Data will be made available on the National Climatic Data Center (https://www.ncdc.noaa.gov/dataaccess/paleoclimatology‐data).
