# Supplementary material for: Revising evidence of hurricane strikes on Abaco Island (The Bahamas) over the last 700 years
Source: Sci Rep. 2020 Oct 6;10:16556. doi: 10.1038/s41598-020-73132-x (PMC7538955; doi:10.1038/s41598-020-73132-x)
Supplement: Supplementary file 1 — Supplementary information [file 41598_2020_73132_MOESM1_ESM.pdf]

SUPPLEMENTAL INFORMATION

Revising evidence of hurricane strikes on Abaco Island (The Bahamas) over the last 700 years

Tyler S. Winkler[1*], Peter J. van Hengstum[1, 2], Jeffrey P. Donnelly[3], Elizabeth J. Wallace[4], Richard M. Sullivan[1], Dana MacDonald[3,5], Nancy A. Albury[6]

1. Department of Oceanography, Texas A&M University, College Station, Texas, 77840.
2. Department of Marine Sciences, Texas A&M University at Galveston, Galveston, Texas, 77554.
3. Department of Geology and Geophysics, Woods Hole Oceanographic Institution, Woods Hole, Massachusetts, 02543
4. Massachusetts Institute of Technology/Woods Hole Oceanographic Institution Joint Program in Oceanography, Woods Hole, Massachusetts 02543, USA
5. Department of Geosciences, University of Massachusetts-Amherst, Amherst, Massachusetts, USA, 01003
6. National Museum of The Bahamas, PO Box EE-15082, Nassau, The Bahamas.

*Corresponding author: Tyler Winkler (tylerwinkler@tamu.edu)

SUPPLEMENTAL 1. Hurricane track density distribution map methods

The map of hurricane track density distribution was based on International Best Track Archive for Climate Stewardship (*IBTrACS) v04* hurricane track segments from 1851 to 2019 CE[1,2]. Hurricane intensity (Saffir-Simpson scale) at each segment is from the IBTrACS v4 subset USA_Agency_SSHS, which is derived from HURDAT_ATL maximum wind speed data. The *USA_Agency* subset is a hierarchical composite of multiple meteorological organizations measured maximum wind speed data, but is primarily populated by HURDAT2 data. For more information on how data from the *USA_Agency* subset was compiled, see Knapp et al.[1,2].

To create this map, a grid of 50x50 km cells was overlaid across the North Atlantic Basin (including the GOM and Caribbean) in *ArcMap v10.7.1* software. *IBTrACS v04* storm tracks were then filtered to exclude any storm track segments that were not ≥cat. 2 (or 3 in **Supplementary Fig. S1**) hurricanes, meaning that segments of storms with maximum wind speeds <154 km hr^{-1} were not counted at any point, even if the storm reached ≥cat. 2 intensity at other points along its track. Once the data was filtered, the storm-tracks were “dissolved” (i.e., merged) into a single track for each qualifying storm using the Storm Identification number (SID) as a qualifier. This left 566 individual storms segments that qualified as ≥cat. 2 hurricanes overall. These 566 hurricane tracks were then spatially joined to the 50x50 km grid cells so that the number of segments that fell within each could be summed. These grid cells were then spatially joined to points located at the center of each cell so that the point could be assigned the z-value of the sum of ≥cat. 2 or 3 storm tracks within the grid cell.

In order to spatially characterize the gradients in storm counts throughout the basin, we utilized Inverse Distance Weighted (IDW) interpolation with the ArcMap *Geostatiscal Analyst* toolbox. This interpolation method allows the user to specify the degree to which surrounding point values influence one another. We utilized a power factor of 8, meaning that the IDW interpolation weighted proximal value points far more heavily that distal points. This is an appropriate method given that hurricanes strikes are relatively localized events and each grid cell, and the point with event count values were richly and evenly disturbed across the north Atlantic. Given that storm tracks are dynamic features that are geographically stochastic (albeit, not necessarily climatologically), IDW interpolation using a higher power factor of 8 will better preserve extremes and spatial count gradients in data values that are evenly spaced and relatively abundant.

The resulting interpolated output was visualized using six in event frequency bins. We acknowledge increased levels of uncertainty in the track and measured intensity of storms that occurred prior to the satellite era of hurricane observation in the 1960s, assuming that observational error was consistent from region to region over time, we can be confident in the results of this spatial analysis as a metric of relative geographic variability in hurricane track density.

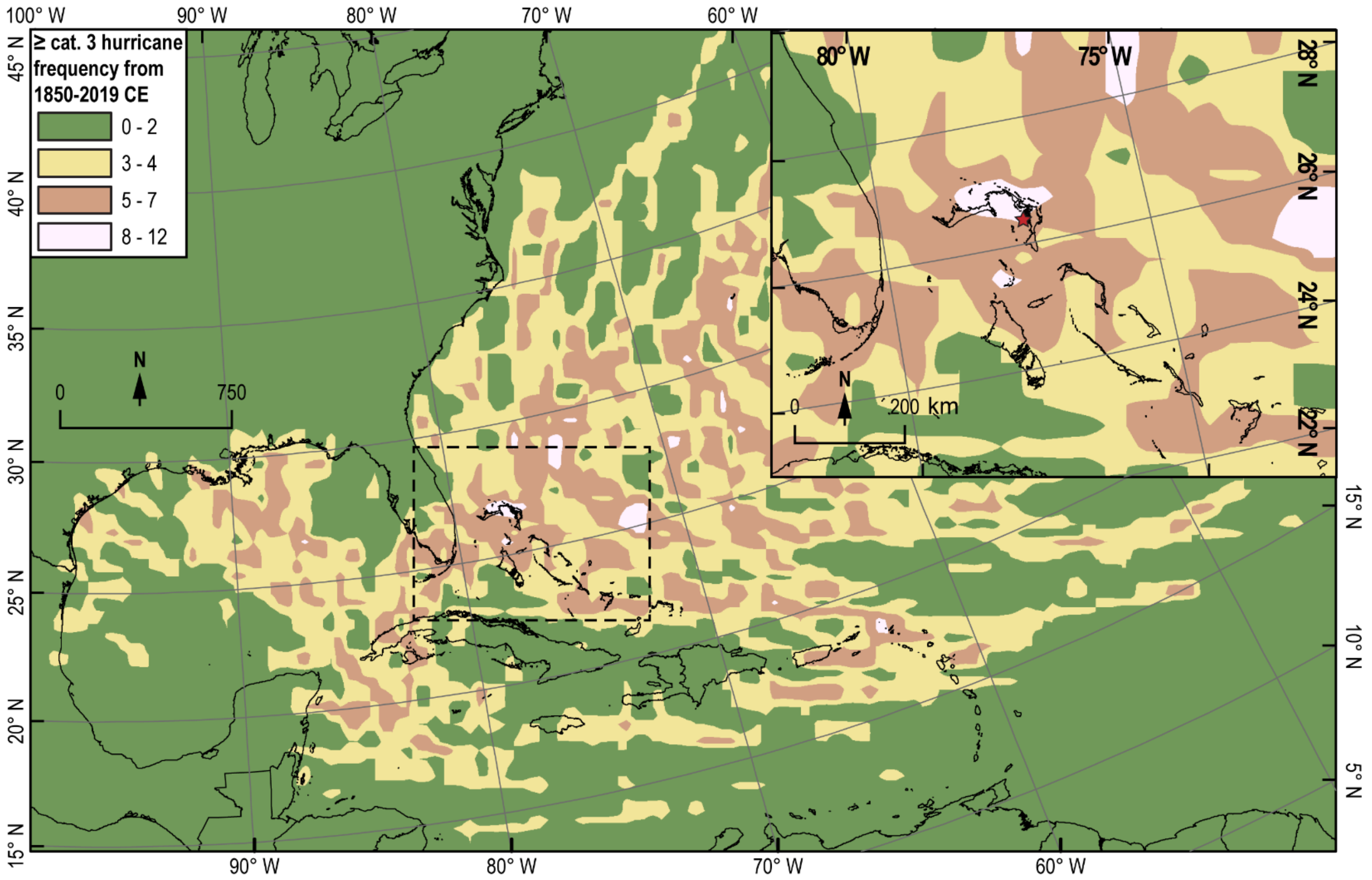

Supplementary Fig. S1| Frequency of exposure to ≥category 3 hurricane winds a 50 km radius from 1850 to 2019 CE throughout the North Atlantic. As with **Fig. 1** from the primary manuscript and described above, but a density map of higher intensity >category 3 events. Storm track and intensity data is from the IBTrACS v04[1,2] dataset. Saffir-Simpson storm intensity is specifically derived from the IBTrACS v4 subset USA_Agency_SSHS, which is derived from HURDAT_ATL maximum wind speed data. Thatchpoint Blue Hole is indicated with a red star. The regions that we refer to as modern hurricane hotspots are pinkish white in color (11-16 ≥category 2 events within 50 km s). Basemaps were downloaded from *DIVA-GIS*[3]. Map and assciated spatial calculations were performed in ArcMap 10.7.1 software using North America Albers Equal Conic Area projected coordinate system[4].

SUPPLEMENTAL 2. Coring locations in Thatchpoint Blue Hole

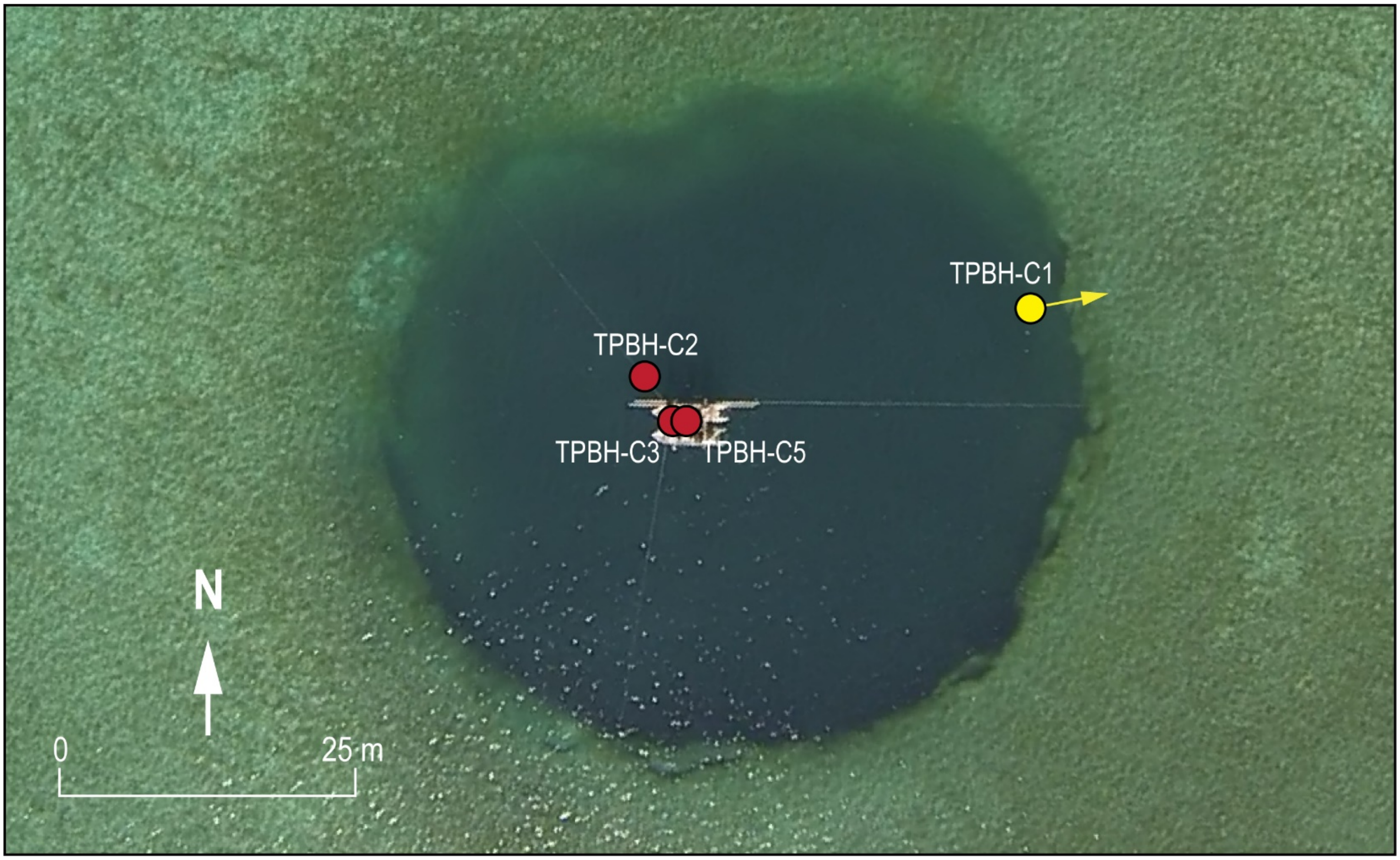

Supplementary Fig. S2| Coring locations in Thatchpoint Blue Hole. The three cores collected in January 2015 (red circles) were TPBH-C2 (26.32342° N, -77.29345° W, 615 cm), TPBH-C3 (26.323390° N, -77.29342° W; recovered length of 836 cm), and TPBH-C5 (26.32339° N, -77.29343° W; 194 cm). These cores were collected using a Rossfelder P3 submersible vibracoring system from the inflatable raft seen in the image. The yellow circle for TPBH-C1 (26.32346° N, -77.29315° W; recovered length of 164 cm) marks the location in which the scientific divers boat was anchored as published by van Hengstum et al. (2014)[5]; however, it is now thought that the is a push core collected on SCUBA in August, 2011 was collected from underneath the wall that overhangs to the east of the yellow circle as marked by the yellow arrow. Aerial imagery was collected by Pete van Hengstum in January 2015 using a DJI Phantom 3 with a mounted GoPro Hero3 digital camera.

SUPPLEMENTAL 3. Age-control points from Thatchpoint Blue Hole composite record.

Supplementary Table S1| Radiocarbon results from Thatchpoint Blue Hole. Of the 16 samples from TPBH-C3 that were radiocarbon dated, only 11 of were included in the Bayesian statistical age model as 5 samples had measured ages older than stratigraphically deeper dates. This suggests that these samples were reworked from older sediments before being redeposited in to TPBH and are not representative of stratigraphic-age. Both dates from TPBH-C2 were included in the age-model. Radiocarbon dates were calibrated using IntCal13 or MarineCal13 calibration curves[6] as described in the **METHODS** in the manuscript. The text "NaN ± NaN" in the "Conventional ^{14}C age" column of sample 1 denotes that the sample is younger than 1950 CE, and was therefore calibrated using the Northern Hemisphere Zone 2 (NHZ2) post-comb calibration data set in CALIbomb[7]. All possible 1σ and 2σ age calibrations are provided for each submitted date.

	Index No.	Accession number	Core	Section depth (cm)	Total core depth (cm)	Material	$F^{14}C$	Conventional ^{14}C age	$d^{13}C$ (‰)	1σ calendar ages in yrs. B_{1950} (probability)	2σ calendar ages in yrs. B_{1950} (probability)	Highest probability 1σ age (cal yrs B_{1950})	Median probability age (cal yrs B_{1950})
TPBH-C3	1	OS-128341	TPBH-C3-1:6	46.5	61.5	Leaf	1.1774 ± 0.0028	NaN ± (NaN)	-26	-39.14 to -37.84 (0.964	-39.84 to -37.77 (0.802	-38.48 ± 1	-38.48
										-37.25 to -37.18 (0.036	-37.46 to -36.94 (0.095		
											-36.36 to -36.24 (0.014		
											-35.93 to -35.83 (0.011		
											-9.13 to -8.50 (0.078)		
	2	OS-137779	TPBH-C3-3:6	37.5	315.5	Unidentifiable Organics	0.922 ± 0.0018	655 ± (15)	-17.04	307-271 (1.000)	364-257 (1.000)	290 ± 20	290
	3	OS-124637	TPBH-C3-3:6	38.5	316.5	Leaf	0.9727 ± 0.0022	225 ± (20)	-25.32	297-284 (0.52)	305-275 (0.504)	290 ± 5	190
										166-154 (0.48)	181-180 (0.004)		
											172-151 (0.417)		
											8-0* (0.075)		
	4	OS-124753	TPBH-C3-3:6	87.5	365.5	Leaf	0.9649 ± 0.0031	285 ± (25)	-25.3	426-392 (0.586)	435-352 (0.613)	410 ± 15	380
										318-298 (0.414)	333-288 (0.387)		
	5	OS-147817	TPBH-C3-4:6	1.5	464.5	Leaf	0.9695 ± 0.002	250 ± (15)	NM	303-291 (1.000)	307-285 (0.926)	295 ± 5	295
											165-157 (0.074)		
	6	OS-127186	TPBH-C3-4:6	100.5	502.5	Leaf	0.9621 ± 0.0019	310 ± (15)	-26.86	426-391 (0.684)	433-355 (0.772)	410 ± 20	395
										387-379 (0.122)	332-306 (0.228)		
										319-309 (0.194)			
	7	OS-124752	TPBH-C3-5:6	22.5	629.5	Unidentifiable Organics	0.9022 ± 0.0019	825 ± (15)	-8.79	486-450 (1.000)	499-428 (1.000)	470 ± 20	465
	8	OS-124638	TPBH-C3-5:6	95.5	702.5	Leaf	0.9525 ± 0.0019	390 ± (15)	-25.75	498-468 (1.000)	503-451 (0.889)	485 ± 15	480
											443-440 (0.011)		
											348-335 (0.1)		
	9	OS-127185	TPBH-C3-6:6	7.5	762.5	Leaf	0.9527 ± 0.002	390 ± (15)	-24.53	498-468 (1.000)	503-451 (0.889)	485 ± 15	480
											443-440 (0.011)		
											348-335 (0.1)		
	10	OS-137463	TPBH-C3-6:6	90.5	845.5	Mollusc	0.8995 ± 0.0017	850 ± (15)	0.01	582-568 (0.253)	603-560 (0.353)	640 ± 25	635
										657-628 (0.747)	664-617 (0.647)		
	11	OS-121213	TPBH-C3-6:6	94.5	849.5	Mollusc	0.8982 ± 0.002	860 ± (20)	1.51	579-570 (0.137)	602-560 (0.274)	645 ± 25	640
										663-630 (0.863)	670-618 (0.726)		
TPBH-C2	12	OS-147909	TPBH-C2-4:6	53.5	390.5	Leaf	0.9294 ± 0.0019	590 ± (15)	-8.79	279-228 (0.961)	282-172 (0.907)	255 ± 25	245
										158-153 (0.039)	168-145 (0.093)		
	13	OS-147910	TPBH-C2-6:6	34.5	560.5	Leaf	0.958 ± 0.0018	345 ± (15)	-26.17	459-429 (0.418)	477-424 (0.401)	445 ± 15	380
										376-348 (0.369)	396-317 (0.599)		
										339-323 (0.213)			
Unused dates	14	OS-128299	TPBH-C3-2:6	56.5	201.5	Mollusc	0.9397 ± 0.0019	500 ± (15)	2.03	186-165 (0.119)	233-47 (0.923)	105 ± 45	115
										146-59 (0.846)	25-0* (0.077)		
										5-0* (0.036)			
	15	OS-137462	TPBH-C3-2:6	88.5	233.5	Mollusc	0.9034 ± 0.0018	815 ± (15)	1.69	481-442 (1.000)	495-421 (1.000)	460 ± 20	460
	16	OS-137762	TPBH-C3-3:6	34.5	312.5	Fish Bone	0.8683 ± 0.0021	1130 ± (20)	-11.23	697-655 (1.000)	726-641 (1.000)	680 ± 20	680
	17	OS-121212	TPBH-C3-3:6	85.5	363.5	Mollusc	0.886 ± 0.0023	975 ± (20)	1.46	570-534 (0.656)	622-518 (1.000)	565 ± 30	565
										597-575 (0.344)			
	18	OS-137780	TPBH-C3-5:6	94.5	701.5	Unidentifiable Organics	0.9303 ± 0.0023	580 ± (20)	-18.67	271-224 (0.702)	278-139 (1.000)	250 ± 25	225
										214-194 (0.181)			
										161-149 (0.118)			

Opaque Spherules and Pollen

Supplementary Table S2| Pollen and Opaque Spherule abundance in TPBH. Pollen in recorded in percentages. Nomenclature follows Correll and Correll [8].

	Core	TPBH-C3-1:6	TPBH-C3-1:6	TPBH-C3-2:6	TPBH-C3-2:6	TPBH-C3-3:6	TPBH-C3-3:6	TPBH-C3-4:6
	**Section depth (cm)**	**51.5**	**124.5**	**61.5**	**130.5**	**58.5**	**127.5**	**44.5**
	**Total core depth (cm)**	**66.5**	**139.5**	**206.5**	**275.5**	**336.5**	**405.5**	**507.5**
	**Age-model median age (yr CE)**	**1976**	**1926**	**1871**	**1814**	**1769**	**1709**	**1622**
Fossil Pollen Concentration	Exotic pollen added (#grains/2.5cm^3)	27,000	27,000	27,000	27,000	27,000	27,000	27,000
	Fossil pollen counted (#grains/2.5cm^3)	103	102	107	102	105	77	101
	Exotic pollen counted (#grains/2.5cm^3)	69	68	102	127	70	147	93
	Fossil pollen concentration (#grains/cm^3)	16121.73913	16200	11329.41176	8674.015748	16200	5657.142857	11729.03226
Opaque Spherules	Raw counts opaque spherules (#spherules/2.5cm^3)	360	925	222	148	34	124	19
	Opaque spherules (#spherules/cm^3)	144	370	88.8	59.2	13.6	49.6	7.6
Mangroves	*Conocarpus erectus* L.	0	0	0	1.92	2.33	6.06	0
	Rhizophora mangle L.	1.19	1.15	0	0	2.33	3.03	3.23
	Avecennia germinans (L.)L.	1.19	0	0	0	0	0	0
Trees & Shrubs	Whole Grains *Pinus caribaea* Morelet	33.33	25.29	37.78	23.86	30.23	22.73	19.35
	1/3 Grains *Pinus caribaea* Morelet	19.05	34.48	33.33	27.27	23.26	22.73	34.41
	Total *Pinus caribaea* Morelet	52.38	59.77	71.11	51.13	53.49	45.46	53.76
	Arecaceae Bercht. and J.Presl, nom. cons.	2.38	10.34	2.22	2.27	3.49	9.09	10.75
	Acacia Mill.	2.38	0	2.2	0	5.81	1.52	1.08
	Metopium toxiferum (L.) Krug and Erb.	0	1.15	0	0	0	3.03	1.08
	Bursera Jacq. ex L.	1.19	0	0	0	0	0	0
	Buxus bahamensis Baker	3.57	0	2.22	4.55	3.49	0	1.08
	Caesalpinoideae D C.	3.57	4.6	2.22	7.95	1.16	4.55	14.58
	Capparis L.	0	0	1.11	1.14	0	0	0
	Coccoloba P. Br. Ex L. nom. cons.; Howard	0	1.15	2.22	0	0	1.52	0
	Cordia L.	0	2.3	2.22	0	0	3.03	0
	Cupressaceae Bartl.	0	1.15	1.11	1.14	0	0	2.15
	Euphorbiaceae Juss.	2.38	0	0	0	0	0	0
	Fabaceae Lindley	0	3.11	1.11	0	1.16	0	0
	Myrica cerifera L.	5.95	2.3	1.11	6.82	0	9.09	1.08
	Myrtaceae Juss.	1.19	1.15	0	1.14	0	0	0
	Picrodendron baccatum (L.) Krug. And Erb	0	0	0	0	3.49	0	3.23
	Randia aculeata L.	3.57	2.3	0	1.14	0	0	1.08
	Swietenia mahogani (L.) Jacq.	0	1.15	0	0	0	0	0
	Tabebuia DC.; Gentry	0	1.96	0	0	0	1.52	0
Herbs & Pteridophytes	*Asteraceae* Dum., nom. cons.	0	0	1.11	1.14	0	0	1.08
	Bromeliaceae Juss.	0	0	0.93	0	0	0	0
	Amarthaceae Juss.	0	0	0	4.55	0	0	0
	Convolvulaceae Juss.	2.38	0	0	0	1.16	0	2.15
	Poaceae Barnhart	3.51	1.15	6.67	7.95	2.33	3.03	4.3
	Batis maritima L.	0	0	0	0	0	0	0.99
	Heliotropium L.	0	1.15	0	0	0	1.52	2.15
	Salicornia L.	0	2.3	1.11	2.27	0	0	0
	Sesuvium L.	0	1.15	0	1.14	0	0	0
	Pteridium (L.) Kuhn	6.8	3.92	12.5	4.9	11.43	5.19	0
	Trilete Spore	0	0	0	0	0	0	5.94
Indeterminates & Unknowns	Indeterminates and Unknowns	23.5	13.42	5.02	13.37	11.43	16.67	7.1

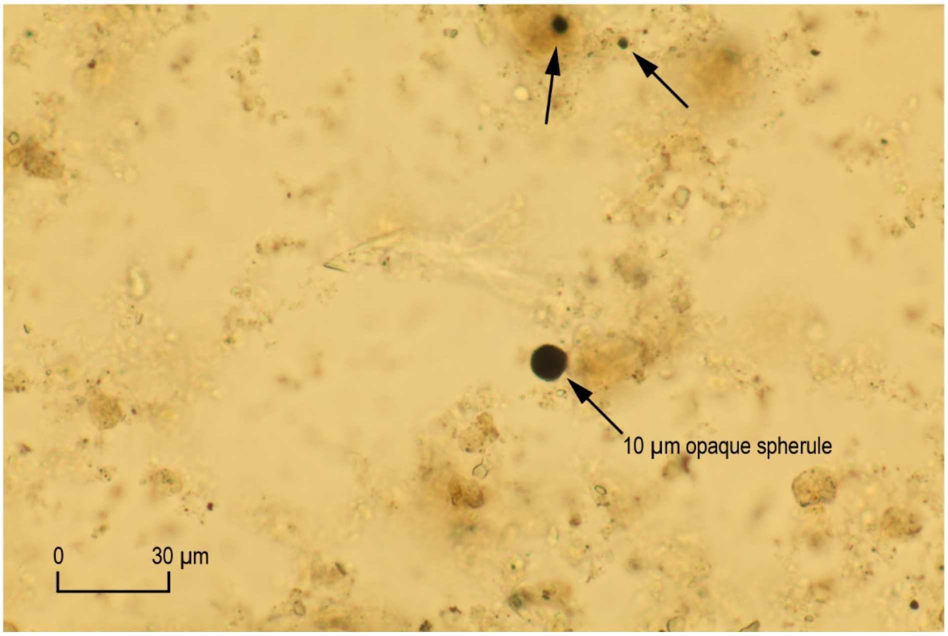

Supplementary Fig. S3| Imagery of opaque spherules from 139-140 cm in the TPBH composite record (TPBH-C3-1:6_124.5 cm). The large spherule near the center of the frame is exactly 10 um in diameter, well within the range of opaque spherule size range) found by Clark and Patterson [9] (5-25 um) , and the broader range of sizes) from Griffin and Goldberg [10] (approximately 4-50 um). The image was taken on an Olympus BH-2 OM with a Cannon DS126201 digital camera in the Hartshorn Quaternary Laboratory at University of Massachusetts Amherst Department of Geosciences.

Total fossil pollen was counted to over 100 grains per level except for TPBH-C3-3:6_127.5 cm, and total fossil pollen concentrations ranged from 16,200 to 5,657 grains/cm^3. At level TPBH-C3-3:6_127.5 cm (400.5 cm total core depth), the lower 5,657 grain concentration counts limited the total counts to 77 grains. Total fossil pollen concentrations were calculated using the method of Bennett and Willis [11]. As discussed in **METHODS** subsection **Age Control**, the sharp peak in opaque spherules at 140 cm (TPBH-C3-1:6_124.5 cm) is interpreted as an artifact of the "Wilson City" logging settlement from 1906 to 1916 CE (due east of TPBH), and possibly a regional increase in economic activity including shipping around the turn of century before late WWI[12].

Gamma Dating: Methods and Results.

Downcore radionuclide activity for ^{137}Cs and ^{210}Pb was measured to better constrain the modern chronology of the upper portion of TPBH-C3. ^{137}Cs is a manmade radionuclide that is useful for identifying the onset of hydrogen thermonuclear weapons testing in 1954 and its moratorium in 1963[13]. To quantify ^{137}Cs activity, bulk sediment was sampled at 5-15 cm intervals, desiccated, and powdered. Powdered samples were then placed on a Canberra GL2020RS low energy Germanium gamma well detector where the gamma decay of the sample was measured for a 24-hour period.

The peak in ^{137}Cs activity associated with 1963 CE moratorium on nuclear weapons testing falls at 80-81 cm. Independent of this constraint, the sedimentary age-model predicts as median age of 1963 CE at 79-80 cm (**Supplementary Fig. S4**). Further, the onset of detectable levels of ^{137}Cs around 1954 CE occurs between 115 to 100 cm in the composite TPBH record. This is within the age and depth error range of the Bayesian age-model (**Fig. 3**), where 1954 CE occurs at a minimum depth of 69 cm, a median depth of 89-90 cm, and maximum depth of 124 cm. Altogether, measure ^{137}Cs provides a useful independent assessment the veracity of the TPBH age-model during the instrumental record and of the TPBH composite record sedimentary age-model that was derived from radiocarbon dates and an opaque spherule chronohorizon.

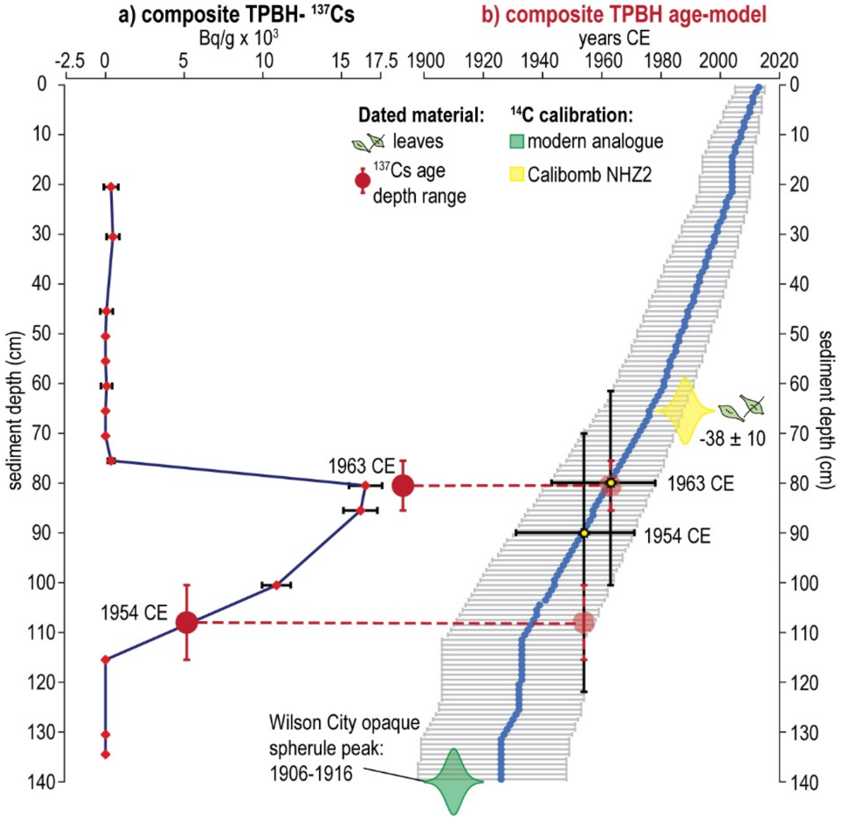

Supplementary Fig. S4| 137Cs activity compared to the radiocarbon-based Bayesian sedimentary age-model. a) Red diamonds represent the sampling locations and measured ^{137}Cs activity in 1000 Becquerels per gram (Bq $-g^{-1}$ x 10^3). The black bars on each diamond show the uncertainty in measured ^{137}Cs activity. Red circles show the estimated 1954 and 1963 CE chronohorizon depths based on the 137Cs activity, and the red error bars show the depth uncertainty on where that peak could have occurred (±5 cm for 1963 CE, ±7.5 cm for 1954 CE onset). **b**) The blue dots represent the median age at each cm interval for the first 140 cm of the TPBH composite record, and the grey error bars represent the 2σ age uncertainty at each interval. The yellow stars represent the depth that the radiocarbon age-model predicts to have median ages of 1954 and 1963 CE, and the black bars show the uncertainty for age (horizontal) and depth at each point.

SUPPLEMENTAL 4. Event Attributions

Supplementary Table S3| Event attributions for event beds with median dates from 1940 to 2015 CE. This table lists all hurricane events within 50 and 115 km of TPBH from 1940 to 2015 CE based on IBTrACS[1,2] and supplements the discussion subsection **Historical Hurricane Strikes: Calibrating the Record** and **Fig. 4**. This portion of the instrumental record has higher confidence due to aircraft monitoring of meteorological events beginning in the 1940s, and the eventual development of satellite monitoring in the 1960s[14,15]. The hurricane(s) that is most likely attributed to the event bed is in bold text. Grey text is used for events that violate the Principle of Superposition if we assume prior attributions are correct, and stars are used to denote hurricanes that are attributed to stratigraphically higher (shallower) event beds.

Event Bed	*Median Age (year CE)*	*2σ Age-Range (year CE)*	*Historical Events within 50 km*	*Passage Details*	*Historical Events within 115 km*	*Passage Details*	*Most Probable Event(s)*
E1	2013	2005-2015	Sandy, 2012 (cat. 1)	45 km E	Noel, 2007 (cat. 1)	80 km E	**Irene, 2011 (cat. 2)**, most intense and proximal of possible events
			**Irene, 2011 (cat. 2)**	**15 km east**			
E2	2004	1995-2011	*Irene, 2011 (cat. 2)	*15 km E	Noel, 2007 (cat. 1)	80 km E	**Jeanne, 2004 (cat. 3), Frances, 2004 (cat. 2)**; high coarse sediment % likely combined result of two events in the same year
			**Jeanne, 2004 (cat. 2)**	**50 km N**	Dennis, 1999 (cat. 1)	60 km NE	
			**Frances, 2004 (cat. 3)**	**40 km S**			
			Floyd, 1999 (cat. 3)	15 km E			
			Erin, 1995 (cat. 1)	25 km S			
E3	1995	1982-2004	*Jeanne, 2004 (cat. 2)	*50 km N	Dennis, 1999 (cat. 1)	60 km NE	**Floyd, 1999 (cat. 3)**; storm surge from this storm had profound impacts across Abaco due to intensity and proximity of this storm
			*Frances, 2004 (cat. 3)	*40 km S	Andrew, 1992 (cat. 4-5)	112 km S	
			**Floyd, 1999 (cat. 3)**	**15 km S**			
			Erin, 1995 (cat. 1)	25 km S			
E4	1986	1972-1996	Erin, 1995 (cat. 1)	25 km S	**Andrew, 1992 (cat. 4-5)**	**112 km S**	**Andrew, 1992 (cat. 4-5)**; in passing NE→SW southward of TPBH, the peak winds from front right quadrant directed water from Atlantic toward TPBH
No event beds	1951-1990				Betsy, 1965 (cat. 3)	70 km E	No significant event beds
					Janice, 1958 (cat. 1)	100 km E	
					Betsy, 1956 (cat. 3)	84 km E	
					Able, 1951 (cat. 1)	90 km NE	
E5	1941	1915-1961	**21 Sep. 1947 (cat. 3)**	**43 km NE**	Janice, 1958 (cat. 1)	100 km E	**21 Sep. 1947 (cat. 3), 12 Sep. 1946 (cat. 1)**; First ≥cat. 2 events within 50 km of TPBH prior the lull in activity from 1950 to 1990 CE
			**12 Sep. 1946 (cat. 1)**	**30 km SW**	Betsy, 1956 (cat. 3)	84 km E	
			5 Sep. 1933 (cat. 3)	15 km W	Able, 1951 (cat. 1)	90 km NE	
			29 Jul. 1933 (cat. 1)	4 km N	11 Aug. 1939 (cat. 1)	65-100 km S	
			6 Sep. 1932 (cat. 5)	Direct strike	4 Nov. 1935 (cat. 2)	55 km NW	
					29 Sep. 1935 (cat. 4)	95 km NW	
					6 Sep. 1934 (cat. 1)	80 km NE	
					3 Sep. 1933 (cat. 4)	75 km S	
					25 Sep. 1929 (cat. 4)	105 km SE	
					16 Sep. 1928 (cat. 4)	115 km SW	
					26 Jul. 1926 (cat. 3)	115 km SW	
					21 Oct. 1926 (cat. 3)	90 km NW	
					27 Sep. 1923 (cat. 1)	100 km E	

Supplementary Table S4| Event attributions for event beds with median dates from 1850 to 1940 CE. This table lists all hurricane events within 50 and 115 km of TPBH from 1850 to 1940 CE based on IBTrACS[1,2] and supplements the attribution text below and **Fig. 4**. This portion of the instrumental record has lower confidence regarding observations of storm intensity, track, translation speed, size, etc.[14,15]. The hurricane(s) that is most likely attributed to the event bed is in bold text. Grey text is used for events that violate the Principle of Superposition if we assume prior attributions are correct, and stars are used to denote hurricanes that are attributed to stratigraphically higher (shallower) event beds.

Event Bed	*Median Age (year CE)*	*2σ Age-Range (year CE)*	*Historical Events within 50 km*	*Passage Details*	*Historical Events within 115 km*	*Passage Details*	*Most Probable Event(s)*
E6	1936	1910-1957	*21 Sep. 1947 (cat. 3)	*43 km NE	Betsy, 1956 (cat. 3)	84 km E	**29 Sep. 1935 (cat. 4)**
			*12 Sep. 1946 (cat. 1)	*30 km SW	Able, 1951 (cat. 1)	90 km NE	
			5 Sep. 1933 (cat. 3)	15 km W	11 Aug. 1939 (cat. 1)	65-100 km S	
			29 Jul. 1933 (cat. 1)	4 km N	4 Nov. 1935 (cat. 2)	55 km NW	
			6 Sep. 1932 (cat. 5)	Direct strike	**29 Sep. 1935 (cat. 4)**	**95 km NW**	
					6 Sep. 1934 (cat. 1)	80 km NE	
					3 Sep. 1933 (cat. 4)	75 km S	
					25 Sep. 1929 (cat. 4)	105 km SE	
					16 Sep. 1928 (cat. 4)	115 km SW	
					26 Jul. 1926 (cat. 3)	115 km SW	
					21 Oct. 1926 (cat. 3)	90 km NW	
					27 Sep. 1923 (cat. 1)	100 km E	
E7	1933	1906-1954	*21 Sep. 1947 (cat. 3)	*43 km NE	Betsy, 1956 (cat. 3)	84 km E	**6 Sep. 1932 (cat. 5)**
			*12 Sep. 1946 (cat. 1)	*30 km SW	Able, 1951 (cat. 1)	90 km NE	
			5 Sep. 1933 (cat. 3)	15 km W	11 Aug. 1939 (cat. 1)	65-100 km S	
			29 Jul. 1933 (cat. 1)	4 km N	4 Nov. 1935 (cat. 2)	55 km NW	
			**6 Sep. 1932 (cat. 5)**	**Direct strike**	*29 Sep. 1935 (cat. 4)	95 km NW	
					6 Sep. 1934 (cat. 1)	80 km NE	
					3 Sep. 1933 (cat. 4)	75 km S	
					25 Sep. 1929 (cat. 4)	105 km SE	
					16 Sep. 1928 (cat. 4)	115 km SW	
					26 Jul. 1926 (cat. 3)	115 km SW	
					21 Oct. 1926 (cat. 3)	90 km NW	
					27 Sep. 1923 (cat. 1)	100 km E	
E8	1926	1898-1948	*21 Sep. 1947 (cat. 3)	*43 km NE	11 Aug. 1939 (cat. 1)	65-100 km S	**25 Sep. 1929 (cat. 4), 16 Sep. 1928 (cat. 4)**
			*12 Sep. 1946 (cat. 1)	*30 km SW	4 Nov. 1935 (cat. 2)	55 km NW	
			5 Sep. 1933 (cat. 3)	15 km W	*29 Sep. 1935 (cat. 4)	95 km NW	
			29 Jul. 1933 (cat. 1)	4 km N	6 Sep. 1934 (cat. 1)	80 km NE	
			*6 Sep. 1932 (cat. 5)	Direct strike	3 Sep. 1933 (cat. 4)	75 km S	
			1 Oct. 1908 (cat. 2)	5 km west	**25 Sep. 1929 (cat. 4)**	**105 km SE**	
					**16 Sep. 1928 (cat. 4)**	**115 km SW**	
					27 Jul. 1926 (cat. 3)	115 km SW	
					21 Oct. 1926 (cat. 3)	90 km NW	
					27 Sep. 1923 (cat. 1)	100 km E	
					12 Oct. 1909 (cat. 2)	75 km W	
					7 Sep. 1906 (cat. 3)	105 km NE	
					1899 (cat. 3)	115 km W	
					1899 (cat. 1)	115 km W	

Supplementary Table S4| Event attributions for event beds with median dates from 1850 to 1940 CE (continued).

Event Bed	*Median Age (year CE)*	*2σ Age-Range (year CE)*	*Historical Events within 50 km*	*Passage Details*	*Historical Events within 115 km*	*Passage Details*	*Most Probable Event(s)*
E9	1918	1890-1942	5 Sep. 1933 (cat. 3)	15 km west	11 Aug. 1939 (cat. 1)	65-100 km S	**1 Oct. 1908 (cat. 2)**
			29 Jul. 1933 (cat. 1)	4 km north	4 Nov. 1935 (cat. 2)	55 km NW	
			*6 Sep. 1932 (cat. 5)	*Direct strike	*29 Sep. 1935 (cat. 4)	95 km NW	
			**1 Oct. 1908 (cat. 2)**	**5 km W**	6 Sep. 1934 (cat. 1)	80 km NE	
			6 Sep. 1896 (cat. 3)	10 km W	3 Sep. 1933 (cat. 4)	75 km S	
			12 Oct. 1893 (cat. 3)	40 km N	*25 Sep. 1929 (cat. 4)	*105 km SE	
			26 Aug. 1893 (cat. 3)	20 km SW	*16 Sep. 1928 (cat. 4)	*115 km SW	
					26 Jul. 1926 (cat. 3)	115 km SW	
					21 Oct. 1926 (cat. 3)	90 km NW	
					27 Sep. 1923 (cat. 1)	100 km E	
					12 Oct. 1909 (cat. 2)	75 km W	
					7 Sep. 1906 (cat. 3)	105 km NE	
					30 Oct. 1899 (cat. 1)	115 km W	
					13 Aug. 1899 (cat. 3)	115 km W	
					22 Oct. 1896 (cat. 2)	75 km NW	
E10	1883	1851-1909	*1 Oct. 1908 (cat. 2)	*5 km W	12 Oct. 1909 (cat. 2)	75 km W	**26 Aug. 1893 (cat. 3)**
			6 Sep. 1896 (cat. 3)	10 km W	7 Sep. 1906 (cat. 3)	105 km NE	
			12 Oct. 1893 (cat. 3)	40 km N	30 Oct. 1899 (cat. 1)	115 km W	
			**26 Aug. 1893 (cat. 3)**	**20 km SW**	13 Aug. 1899 (cat. 3)	115 km W	
			22 Aug. 1887 (cat. 3)	25 km N	22 Oct. 1895 (cat. 2)	75 km NW	
			9 Sep. 1883 (cat. 2)	40 km W	17 Aug. 1879 (cat. 1)	55 km E	
			2 Oct. 1866 (cat. 4)	30 km W	24 Aug. 1871 (cat. 2)	110 km S	
					15 Aug. 1871 (cat. 3)	55 km N	
					12 Oct. 1870 (cat. 1-2)	60 km W	
E11	1873	1841-1900	6 Sep. 1896 (cat. 3)	10 km W	30 Oct. 1899 (cat. 1)	115 km W	**2 Oct. 1866 (cat. 4)**
			12 Oct. 1893 (cat. 3)	40 km N	13 Aug. 1899 (cat. 3)	115 km W	
			*26 Aug. 1893 (cat. 3)	*20 km SW	22 Oct. 1895 (cat. 2)	75 km NW	
			22 Aug. 1887 (cat. 3)	25 km N	17 Aug. 1879 (cat. 1)	55 km E	
			9 Sep. 1883 (cat. 2)	40 km W	24 Aug. 1871 (cat. 2)	110 km S	
			**2 Oct. 1866 (cat. 4)**	**30 km W**	15 Aug. 1871 (cat. 3)	55 km N	
					12 Oct. 1870 (cat. 1-2)	60 km W	

This section expands the event attributions of hurricane events in the instrumental record to coarse-sediment in the TPBH composite record from 1940 to 1850 CE. This early portion of the instrumental record has higher observational uncertainty as it precedes the use of satellites and aircraft to monitor hurricanes[14,15]. The event bed with the highest coarse-sediment fraction between 1950 to 1901 CE (E7) has a median age of 1933 (2σ age-range: 1954 to 1906 CE) and was likely deposited by the 1932 hurricane which passed directly over TPBH as a category 5 storm on 6 September 1932. E6 above has a median age of 1936 CE (2σ age-range: 1957 to 1910 CE) and was potentially deposited by one, or several, storms that impacted Abaco between 1933 and 1935. On 29 September 1935 CE, a category 4 hurricane passed ~100 km northwest of TPBH. Three storms impacted Abaco in 1933 CE: (i) a category 1 storm passed directly over TPBH traveling from east to west across Abaco on July 29, (ii) a category 4 storm passed about 80 km southwest of TPBH on September 3, and (iii) a category 3 storm that passed over the site on October 5 (center of circulation passing about 10 km north) while transiting southwest to northeast across Abaco (**Fig. 4d**). This trajectory of the 5 October 1933 event would put TPBH on the more intense quadrant of the storm with the strongest winds focused onshore. A weak category 1 hurricane passed to ~65 km east of the site on 6 September 1934 CE, but it likely did not generate event beds E6 or E7

because of its distal passage and weak intensity. Two additional hurricanes impacted Abaco in 1935 CE. On 29 September 1935 CE a category 4 storm passed about 95 km to the northwest of TPBH, and on November 4 a category 2 storm passed about 40 km north. While it is not possible to rule out contributions from the three hurricanes in 1933 to these deposits, we suggest that E6 is attributable to the distal 1935 category 4 hurricane and E7 is largely attributable to the 1932 category 5 hurricane that was a direct hit on TPBH. This distinction is made given the 4 cm interval of relatively finer sediments between these peaks in the composite record (108 to 113 cm), which would represent ~5 years of time given he average sedimentation rate of 1.3 cm yr^{-1}.

Event bed E8 has a median age of 1926 CE (2σ age-range: 1948 to 1898 CE). Assuming we have correctly attributed the 1933 CE and 1935 CE hurricanes to event bed E6, and the 1932 category 5 hurricane to E7, five other hurricanes could be responsible for this bed (**Fig. 4d**). Two hurricanes passed ~115 km to the southwest of TPBH on similar southeast to northwest trajectories, the first passing on 27 July 1926 as a category 3 storm and the second on 16 September 1928 at category 4 strength. On 25 September 1929 a category 4 storm passed ~105 km south of the site on a northwest to southeast trajectory. Peak winds during the closest passage of the September 1928 and 1929 category 4 hurricanes to TPBH would have been directed offshore, potentially limiting high-magnitude storm surge for transporting sediment. A category 3 hurricane passed from southwest to northeast about 90 km north of TPBH on 21 October 1926, following a path that would have directed peak onshore winds toward TPBH, but no other event bed can be clearly attributed to a category 3 event outside of a 50 km range. Given that we have attributed deposit E4 to Hurricane Andrew, which had a similar track as the September 1928 and 1929 category 4 hurricanes, we attribute this event to one of these storms, but acknowledge a high-degree of uncertainty surrounding this attribution. Event bed E9 has a median age of 1918 (2σ age-range: 1890-1942). If our attributions of event beds E7 and E8 are correct, we can eliminate the storms in the 1920s as contributing to deposit E9. A category 2 hurricane that passed directly over TPBH on 1 October 1908 is the most likely candidate for this deposit.

Event beds E10-11 were deposited in the last half of the 19th century, which corresponds with the greatest uncertainties in the sedimentary age-model during the historical period (2σ age: ±28 years, n = 62, **Fig. 4a**) and the observational dataset[48-51] (precision/accuracy of storm track coordinates and intensity measurements). As such, we consider the attributions made from 2014 to 1900 CE to be far more reliable for characterizing the sensitivity of the composite record. With this in mind, event bed E10 has a median age of 1883 (2σ age-range: 1909 to 1851 CE) and event bed E11 has a median age of 1873 (2σ age range: 1900 to 1881 CE). 14 storms passed within 115 km of TPBH between 1855 and 1899 CE (**Fig. 4e**), but only three were likely to deposit event beds. Three of these events passed about 40 km west of the TPBH on south to north trajectories on 26 August 1893, 9 September 1883, and 2 October 1866 as category 3, 2, and 4 storms, respectively. We attribute event bed E10 to the 1893 hurricane given that it was more intense than the 1883 event, and event bed E11 to the category 4 hurricane in 1866 CE. The only other intense storms to make a close passage to TPBH were three category 3 events that all passed ~20 to ~40 km northeast of the site (1896 CE, 1893 CE, and 1887 CE; **Fig. 4e**). However, peak winds from storms following this trajectory were likely from the north meaning that TPBH was likely shielded by Abaco Island, thus inhibiting event bed deposition.

References

1 Knapp, K. R., Kruk, M. C., Levinson, D. H., Diamond, H. J. & Neumann, C. J. The international best track archive for climate stewardship (IBTrACS) unifying tropical cyclone data. *Bulletin of the American Meteorological Society* **91**, 363-376 (2010).

2 Knapp, K. R., Diamond, H. J., Kossin, J. P., Kruk, M. C. & Schreck, C. J. III. International Best Track Archive for Climate Stewardship (IBTrACS) Project, Version 4. [Atlantic Basin US_SSHS subset]. NOAA National Centers for Environmental Information. https://doi.org/10.25921/82ty-9e16 [access date: 24 Jan. 2020].

3 Hijmans, R. J., Riojas, E., O'Brien, R., Cruz, M. DIVA-GIS Version 7.5: Countries- Administrative Boundaries. [access date: 24 Jan. 2020]. http://www.diva-gis.org/gdata

4 Snyder, J. P. Equidistant conic map projections. *Annals of the Association of American Geographers* **68**, 373-383 (1978).

5 van Hengstum, P. J. *et al.* Heightened hurricane activity on the Little Bahama Bank from 1350 to 1650 AD. *Continental Shelf Research* **86**, 103-115 (2014).

6 Reimer, P. J. *et al.* IntCal13 and Marine13 radiocarbon age calibration curves 0–50,000 years cal BP. *Radiocarbon* **55**, 1869-1887 (2013).

7 Reimer, P. J., Brown, T. A. & Reimer, R. W. Discussion: reporting and calibration of post-bomb 14C data. *Radiocarbon* **46**, 1299-1304 (2004).

8 Correll, D. S. & Correll, H. B. Flora of the Bahama archipelago. *Flora of the Bahama Archipelago.* (1982).

9 Clark, J. S. & Patterson, W. A. Pollen, Pb-210, and opaque spherules; an integrated approach to dating and sedimentation in the intertidal environment. *Journal of Sedimentary Research* **54**, 1251-1265 (1984).

10 Griffin, J. J. & Goldberg, E. D. The fluxes of elemental carbon in coastal marine sediments. *Limnology and Oceanography* **20**, 456-463 (1975).

11 Bennett, K. D. & Willis, K. J. in *Tracking Environmental Change Using lake Sediments. Volume 3: Terrestrial, Algal, and Siliceous Indicators* (eds J.P. Smol, H.J. Birks, & W.M. Last) Ch. 2, 19 (Kluwer Academic Publishers, 2001).

12 Craton, M. & Saunders, G. *Islanders in the Stream: A History of the Bahamian People. Volume Two: From the Ending of Slavery to the Twenty-First Century*. (JSTOR, 2002).

13 Pennington, W., Cambray, R. S. & Fisher, E. M. Observations on lake sediments using fallout 137Cs as a tracer. *Nature* **242**, 324-326 (1973).

14 McAdie, C., Landsea, C., Neumann, C. J., David, J. E. & Blake, E. S. *Tropical Cyclones of the North Atlantic Ocean, 1851-2006: With 2007 and 2008 Track Maps Included*. Vol. 6 (US Department of Commerce, National Oceanic and Atmospheric Administration, 2009).

15 Vecchi, G. A. & Knutson, T. R. Estimating annual numbers of Atlantic hurricanes missing from the HURDAT database (1878–1965) using ship track density. *Journal of Climate* **24**, 1736-1746 (2011).
